# The ncRNA-mediated regulatory networks of *defensins* and *lysozymes* in *Riptortus pedestris*: involvement in response to gut bacterial disturbances

**DOI:** 10.3389/fmicb.2024.1386345

**Published:** 2024-05-17

**Authors:** Yipeng Ren, Siying Fu, Wenhao Dong, Juhong Chen, Huaijun Xue, Wenjun Bu

**Affiliations:** ^1^Institute of Entomology, College of Life Sciences, Nankai University, Tianjin, China; ^2^Tianjin Key Laboratory of Food and Biotechnology, School of Biotechnology and Food Science, Tianjin University of Commerce, Tianjin, China

**Keywords:** *Riptortus pedestris*, antimicrobial peptides, noncoding RNAs, gut bacterial microbiota, pest control

## Abstract

Insects depend on humoral immunity against intruders through the secretion of antimicrobial peptides (AMPs) and immune effectors via NF-κB transcription factors, and their fitness is improved by gut bacterial microbiota. Although there are growing numbers of reports on noncoding RNAs (ncRNAs) involving in immune responses against pathogens, comprehensive studies of ncRNA-AMP regulatory networks in *Riptortus pedestris*, which is one of the widely distributed pests in East Asia, are still not well understood under feeding environmental changes. The objective of this study employed the whole-transcriptome sequencing (WTS) to systematically identify the lncRNAs (long noncoding RNA) and circRNAs (circular RNA) and to obtain their differential expression from the *R. pedestris* gut under different feeding conditions. Functional annotation indicated that they were mainly enriched in various biological processes with the GO and KEGG databases, especially in immune signaling pathways. Five *defensin* (four novel members) and eleven *lysozyme* (nine novel members) family genes were identified and characterized from WTS data, and meanwhile, phylogenetic analysis confirmed their classification. Subsequently, the miRNA–mRNA interaction network of above two AMPs and lncRNA-involved ceRNA (competing endogenous RNA) regulatory network of one *lysozyme* were predicted and built based on bioinformatic prediction and calculation, and the expression patterns of differentially expressed (DE) *defensins*, and DE *lysozymes* and related DE ncRNAs were estimated and selected among all the comparison groups. Finally, to integrate the analyses of WTS and previous 16S rRNA amplicon sequencing, we conducted the Pearson correlation analysis to reveal the significantly positive or negative correlation between above DE AMPs and ncRNAs, as well as most changes in the gut bacterial microbiota at the genus level of *R. pedestris*. Taken together, the present observations provide great insights into the ncRNA regulatory networks of AMPs in response to rearing environmental changes in insects and uncover new potential strategies for pest control in the future.

## 1 Introduction

Noncoding RNAs (ncRNAs), including transfer RNA (tRNA), ribosomal RNA (rRNA), microRNA (miRNA), long noncoding RNA (lncRNA), circular RNA (circRNA), and so on, do not directly translate into functional proteins, but they play essential roles in gene expression at post-transcriptional level, accounting for up to 70% of the human genome (Palazzo and Lee, 2015). To date, it has been confirmed that ncRNAs, previously regarded as junk molecules, involved in multiple cellular physiological and pathological processes, such as metabolism and apoptosis, with rapidly development of whole-transcriptome sequencing (WTS), which can produce massive data on ncRNAs and mRNAs and predict related regulatory networks based on high-throughput sequencing technology (Cech and Steitz, 2014). Concretely, lncRNAs are long intergenic noncoding RNAs (lincRNAs) longer than 200 nucleotides (nt), and they are necessary for various biological processes, such as cell proliferation, differentiation and development, comprising sense lncRNAs, bidirectional lncRNAs, intronic lncRNAs, and intergenic lncRNAs, by controlling the expression level of the target genes at transcriptional or post-transcriptional levels through *cis* and *trans* regulation (Guttman et al., 2011; Batista and Chang, 2013; Quinn and Chang, 2016; Kopp and Mendell, 2018). On the other hand, circRNAs, first identified in viroids in 1976, are a novel kind of endogenous ncRNA and covalently closed RNA molecules without 5′ caps and 3′ tails (Sanger et al., 1976; Memczak et al., 2013). Four types of circRNAs were classified, containing circular exonic RNAs, intergenic circRNAs, circular intronic circRNAs, and exon–intron circRNAs, and they have a role in the regulation of gene expression by functioning as miRNA sponges, miRNA reservoirs, and linear mRNA splicing competitors (Hansen et al., 2013). Importantly, there are a growing number of reports on both lncRNAs and circRNAs forming competing endogenous RNAs (ceRNAs) by binding to miRNAs, which could degrade or suppress the expression of mRNA in animals (Dykes and Emanueli, 2017).

In invertebrates, the immune system can protect the body from pathogenic infection, of which innate immunity, belonging to nonspecific immunity, is the essential first line of defense against pathogens and parasites. The cellular and humoral immunity, constituting innate immunity in insects, include the encapsulation and phagocytosis of pathogens and the secretion of antimicrobial peptides (AMPs), respectively (Hillyer, 2016; Rolff and Schmid-Hempel, 2016). AMPs are gene-encoded cationic polypeptides, most of which can produce direct antimicrobial effects against bacteria and fungi (Ganz, 2003; Feng et al., 2020). There are many reports and databases that have reported numerous insect-derived AMPs from several families, such as cecropins, defensin, drosocin, drosomycin, diptericins, metchnikowin, attacins, cecropins, and moricins (Staczek et al., 2023). Moreover, it has been found that they are induced by NF-κB transcription factors, including *Dorsal*, *DIF* (dorsal-related immunity factor) and *Relish*, which are mainly controlled by the Toll and Imd signaling pathways in *Drosophila* and other model insects (Myllymaki et al., 2014; Valanne et al., 2022). In brief, defensins (Def) possess many β-sheets and a framework of disulfide-linked cysteines and are divided into three subclasses based on their molecular characterization (Ganz, 2004). Lysozymes (Lyz) are key immune effectors of the innate immune system that protect the host from pathogen infection and exert essential functions in digestion and reproduction (Xiao et al., 2023). Surprisingly, the expression of *defensins* and *lysozymes* was also found to be induced after viral infection in flies and silkworms (Feng et al., 2020). Accumulating evidence has suggested that lncRNAs and circRNAs play significant roles in insect immunity; for example, it has been shown that lncRNA_XR209691.3 can promote the proliferation of BmNPV (*Bombyx mori* nucleopolyhedrosis virus) through interaction with BmHSP70, and the circ_0001432–miR-2774c/miR-3406-5p–mRNA778467/101745232 axis exists in the fat body during BmNPV infection in *Bombyx mori* (Yin et al., 2021; Lin et al., 2023). In *Drosophila*, the lncRNAs CR11538, CR46018 and CR33942 play positive or negative roles in the transcription of immune effector genes in the Toll or Imd pathways (Zhou et al., 2021a,b, 2022a,b). Zhang J. et al. (2020) demonstrated that the expression of circRNAs and bound miRNAs significantly changed during rice black-streaked dwarf virus infection in the *Laodelphax striatellus* gut. However, the transcriptome-wide identification, annotation and prediction of lncRNA– and circRNA–mediated networks of AMPs in heteropteran insects are limited, which could be essential for understanding their adaptive homeostasis in response to environmental changes.

The insect gut is an important channel for pathogen infection, so it is the first line of defense against ingested pathogens and the entry portal for viruses and parasites owing to its connection to the host and external environment (Qiao et al., 2023). Importantly, gut microbial communities exist in host specialized guts and cell bacteriocytes and benefit host survival, homeostasis, development and functioning; thus, the hologenome concept has been inspired, either transiently or tightly, by host–microbe associations, meaning that the gut microbiome has an important role in the host’s physiology and immunity, while microbiome composition may be affected by host selection in response to environmental pressures or changes (Engel and Moran, 2013; Kokou et al., 2018; Fan and Pedersen, 2021). It is recognized that an imbalance in gut bacterial composition may lead to host fitness loss and increase susceptibility to pathogens and metabolic diseases because overall host well-being benefits from gut flora health (Evariste et al., 2019; Mougin and Joyce, 2023). The bean bug, *Riptortus pedestris* (Hemiptera: Alydidae), is a major agricultural pest that is widely distributed in East Asia, such as South Korea, Japan, and China, resulting in significant yield losses and low seed quality of soybean (*Glycine max*), which is an important oil and cash crop (Shan et al., 2021; Wei et al., 2023). Evaluation of the associations between bean bugs and gut bacteria was performed in *R. pedestris* (Jang et al., 2023; Wei et al., 2023; Xia et al., 2023). For example, a specific gut bacterial symbiont, *Caballeronia insecticola* (*Burkholderia insecticola*), promoted the proliferation of stem cells and simultaneously inhibited apoptosis at the tip of gut crypts in *R. pedestris* (Jang et al., 2023). Besides, the knockdown of *thanatin* genes significantly increased the population of gut symbiont *Burkholderia* in the M4 crypt under *Escherichia coli* K12 injection (Lee et al., 2022). *Serratia marcescens* can stably colonize the *R. pedestris* midgut by degrading serralysin toxin by detoxification activity from host gut symbionts (Lee and Lee, 2022). In the present work, we further assumed that shifts in the gut bacteria would affect the expression profiles of AMPs and related ncRNAs in *R. pedestris* under different environments.

To achieve our research goals, twelve WTS libraries from the whole gut of *R. pedestris* were constructed and sequenced under different feeding conditions, producing massive clean reads after quality control and filtration. Of these reads, we identified and investigated the functional, expression and target characterization of lncRNAs and circRNAs by bioinformatic analysis. Furthermore, different numbers of *defensin* and *lysozyme* family genes were screened, and we illustrated their molecular characteristics and evolutionary relationships in insects. Next, a miRNA– *defensin*/*lysozyme* target network and lncRNA*-*mediated ceRNA network of one *lysozyme* were built, and of these ncRNAs in ceRNA network, differential expression was identified and shown based on WTS data. Finally, our results provide data on associations of differentially expressed (DE) *defensins*, DE *lysozymes* and related ncRNAs, as well as most changes in the gut bacterial microbiota at the genus level with Pearson correlation analysis among the different comparison groups. Taken together, the present findings will shed light on future observations on innate immune ncRNAs and AMPs in insects and lay the foundation for pest management in hemipterous pests.

## 2 Materials and methods

### 2.1 Sample collection and treatments

The animal study and the sampling procedures followed the guidelines of the Animal Care and Use Committee of Nankai University. In the current study, all samples of *R. pedestris* were collected from soybean fields at Nankai University, Tianjin, P.R. China, and maintained in climatic chambers at 25 ± 2°C temperature, 60% ± 5% relative humidity, and a 16 h:8 h light/dark photoperiod cycle. The newly hatched eggs from above collected samples were cultured in Petri dishes, and then newly 2nd-instar nymphs were divided into three experimental groups until to emerging adults. In brief, the groups were set as follows: (i) feeding soybean seeds and ascorbic acid (DWA) water (Blank group, B); (ii) feeding potted soybean plants and DWA water (Control group, C); (iii) alternatively feeding with soybean seeds and 0.05% (w/v) DWA or only distilled water containing 50 μg/mL tetracycline (Antibiotic group, A) on the following day (Ren et al., 2023). Later, the whole guts of these above adults and field-collected adults (Field group, F) were dissected (starved after 2 days), and ultimately, three guts from the same group were surface-sterilized in 70% ethanol for 1 min and randomly pooled as one biological replicate, with three biological replicates prepared for each experimental group, and then all these samples were flash-frozen in liquid nitrogen and stored at −80°C until further extraction.

### 2.2 Total RNA extraction and library construction and sequencing

All gut samples were ground separately in liquid nitrogen, and total RNA was isolated using TRIzol reagent (Invitrogen, United States) according to the manufacturer’s instructions. The integrity, quality and purity of extracted RNA were evaluated using 1% agarose gel electrophoresis staining and the A260/A280 ratio by a Bioanalyzer 2,100 system (Agilent Technologies, CA, United States) and a NanoPhotometer® spectrophotometer (IMPLEN, CA, United States), respectively. In this study, the remaining total RNA after library construction was used for qRT–PCR validation.

For lncRNA sequencing, based on the manufacturer’s instructions, ribosomal RNAs (rRNAs) were removed from total RNA using the rRNA Removal Kit (Epicenter, United States) to retain mRNAs and ncRNAs, which were amplified with random hexamer primers to synthesize first-strand cDNA. Next, the second strands were produced by adding buffer, DNA polymerase I, dNTPs, and RNase H. Meanwhile, for circRNA sequencing, above remaining RNA was randomly interrupted into 140–160 nt followed by reverse transcription and amplification with random primers. The above double-stranded cDNA fragments were purified with the AMPure XP system, and the quality of constructed libraries was estimated through the Agilent Bioanalyzer 2,100 system in order to meet the requirements for further sequencing. Lastly, all the prepared libraries were sequenced by the MGI2000 platform (BGI, Wuhan, China) to generate raw reads. In addition, the raw data were submitted to the NCBI Sequence Read Archive database under accession number PRJNA638739.

### 2.3 Data processes and identification of lncRNAs and circRNAs

First, raw reads with adaptor contamination, insert tags, and low-quality and undetermined data were removed to produce clean data using SOAPnuke software (version 1.5.2) (Chen et al., 2018), and simultaneously, the Q20, Q30, and GC contents of the clean data were statistically calculated. Next, the filtered clean reads with high quality were stored in FASTQ format and were mapped to the *R. pedestris* reference genome (accession numbers: SRR12977074) using HISAT2 (version 2.0.4) (Kim et al., 2019; Huang et al., 2021). Transcripts were assembled from the mapped reads of each group using StringTie (version 1.0.4) and were determined locations and interactions using Cufflinks (version 2.2.1) (Pertea et al., 2015).

Second, to classify the protein-coding or noncoding sequences, if the transcripts could be matched in the Pfam database, they were known as mRNAs; otherwise, they were served as lncRNAs. The coding ability of transcripts was further predicted using the Coding-Non-Coding-Index (CNCI; CNCI_threshold >0 is mRNA, CNCI_threshold <0 is lncRNA), Coding Potential Calculator (CPC, version 0.9-r2; CPC_threshold >0 is mRNA, CPC_threshold <0 is lncRNA) and txCdsPredict (txCdsPredict_threshold >500 is mRNA, txCdsPredict_threshold <500 is lncRNA) (Zhang et al., 2023).

Third, Find_circ (version 1.2) and CIRI (version 2.0.5) were employed to identify circRNAs with default parameters (Memczak et al., 2013; Gao et al., 2015). CircRNAs were assigned into all the sequencing groups based on their transcriptional location in the genome and then were classified into exonic, intronic, sense, antisense and intergenic circRNAs (Shen et al., 2017).

### 2.4 Expression analysis of lncRNAs and circRNAs

To investigate the expression patterns of lncRNAs, circRNAs and mRNAs, FPKM (Fragments Per Kilobase of exon model per Million mapped reads) was used to estimate their expression abundance among all the sequencing samples. The Benjamini–Hochberg correction procedure was applied to adjust the resulting *p* values to decrease false discovery rates (FDR). The expression profiles of lncRNAs and circRNAs were calculated and normalized to transcripts per million (TPM), and the significantly differentially expressed lncRNAs (DELs) and circRNAs (DECs) were considered with both absolute log_2_ |Fold Change| >1 and *p* < 0.001 using DESeq R package (version 3.0.3) and Bowtie2 (version 2.2.5) (Love et al., 2014).

### 2.5 Target prediction and bioinformatic analyses

To predict the target genes of lncRNAs, the regions 0–10 kb (kilobase) upstream or 0–20 kb downstream of *cis*–acting lncRNAs were scanned to find co-located target genes, and meanwhile, the lncRNAs were considered trans–acting on the target genes once the binding energy was < −30 and exceeded the above region based on the *cis*/trans-regulatory algorithms (Pearson correlation coefficient ≥ 0.6 and Spearman correlation coefficient ≥ 0.6). In addition, the overlaps between lncRNAs and target genes were classified into four categories: overlap, anti-overlap, incomplete, and anti-incomplete. For co-localization analysis, we searched the protein-coding genes located between 10 kb and 20 kb from the lncRNA and examined the correlation between the expression patterns of each gene and lncRNA (Zhang et al., 2022). To identify the circRNA targeting miRNAs, RNAhybrid (version 0.1) and miRanda (version 3.3a) were employed to predict miRNA sites in circRNAs using the default parameters (Energy < −30) according to Pearson correlation coefficients ≥0.6 and *p* < 0.05 (Sai et al., 2018).

DELs and their target genes, and DECs were blasted to the GO (Gene Ontology) and KEGG (Kyoto Encyclopedia of Genes and Genomes) pathway databases using GOseq R package and KOBAS software, respectively, and they were significantly enriched based on a corrected *p* < 0.01 (Boyle et al., 2004). In addition, the top 20 significantly enriched KEGG pathways were selected and performed among all the comparison groups.

### 2.6 Identification, characterization and phylogenetic analysis of *defensins* and *lysozymes*

The *defensin* and *lysozyme* family genes were identified from WTS data and then were compared to those of other insects by the BLAST program (TBLASTN and BLASTP databases with E-values <0.001; https://www.ncbi.nlm.nih.gov/). Subsequently, the retrieved cDNA sequences were translated using the ExPASy-Translate tool.^1^ The putative domains and signal peptides of all of the above translated protein sequences were predicted with InterProScan^2^ and TMHMM Server (version 2.0; http://www. Cbs.dtu.dk/services/TMHMM), and SignalP (version 5.0; https://services.healthtech.dtu.dk/services/SignalP-5.0/), respectively. The schematic diagrams of the protein structures were drawn and visualized using IBS software (Illustrator for Biological Sequences; version 2.0; https://ibs.renlab.org/#/home). The molecular weights (MWs), isoelectric points (PIs), and grand average of hydropathicity (GRAVY) of amino acid sequences were analyzed by the ExPASy ProtParam tool^3^ (Ren et al., 2022; Xie et al., 2022). The protein structure of the three-dimensional (3D) model was constructed and predicted using SWISSMODEL^4^ and PSIPRED (version 4.0; http://bioinf.cs.ucl.ac.uk/psipred/) (Duvaud et al., 2021; Paysan-Lafosse et al., 2023). The amino acid sequences of defensins and lysozymes from other insects were searched and downloaded from the NCBI GenBank database, and multiple sequence alignments were executed using ClustalW (version 2.1). Finally, two phylogenetic trees were generated using MEGA (Molecular Evolutionary Genetics Analysis; version 9.0) with the neighbor-joining (NJ) method (Kumar et al., 2016), whose statistical supports were calculated with 1,000 bootstrap replicates (Supplementary Table S4), and the results were visualized and optimized using FigTree software (version 1.4.4) (Tong et al., 2015).

### 2.7 The identification and construction of interaction networks

LncRNAs and circRNAs with miRNA binding sites, serving as miRNA sponges, can compete with mRNAs for interaction with target miRNAs involving in the expression regulation of corresponding mRNAs (Hansen et al., 2013). In this study, we constructed a ceRNA network according to the ceRNA hypothesis using Cytoscape software (version 3.9.1). In brief, we selected lncRNAs, circRNAs, miRNAs and mRNAs to build the ceRNA network based on the same expression trends of lncRNAs or circRNAs and mRNAs and opposite expression of those with miRNAs, and the *p* value and |Pearson correlation coefficients| of lncRNA–miRNA, circRNA–miRNA, and miRNA–mRNA pairs were less than 0.05 and more than 0.6, respectively. In addition, the prediction and annotation of the miRNA–mRNA pairs were from our previous report (Ren et al., 2023).

### 2.8 Correlated analysis of the WTS and 16S rRNA sequencing data

To detect the associations between DE *defensin*, DE *lysozyme*s and related DE ncRNAs, and most changes in the gut bacterial microbiota at the genus level among the six comparison groups, the heatmaps were produced using R software (version 2.15.3) based on Pearson correlation coefficients and the levels of statistically significant difference at *p < 0.05, **p < 0.01, and *** *p* < 0.001 (Ren et al., 2023).

### 2.9 Corroboration of WTS data by qRT–PCR

The remaining total RNA after library construction was used to synthesize first strand cDNA with the TransScript II One-Step gDNA Removal and cDNA Synthesis Kit (TransGen, Beijing, China). The quantitative real–time PCR (qRT–PCR) assay was executed with a Green qPCR SuperMix Kit (TransGen, Beijing, China) on a CFX96 real–time PCR detection system (Bio-Rad Laboratories, Hercules, CA) following the manufacturer’s instructions. The PCR procedures were performed as described previously (Fu et al., 2021; Ren et al., 2023). The EF–1α gene was used as an internal control for normalization of the expression levels, and specific primers of selected DE AMPs and ncRNAs were designed by Primer Premier 5 software (Supplementary Table S5). Finally, the results were calculated and displayed between the log_2_ (Fold Change) values from the WTS data and the log _2_ of the relative quantification (RQ) values from above selected AMPs and ncRNAs using qRT**–**PCR reaction (Livak and Schmittgen, 2001), and column charts were produced using GraphPad Prism (version 9.0; GraphPad Software Inc., United States).

## 3 Results

### 3.1 Overview and filtration of WTS data

**Figure 1:**
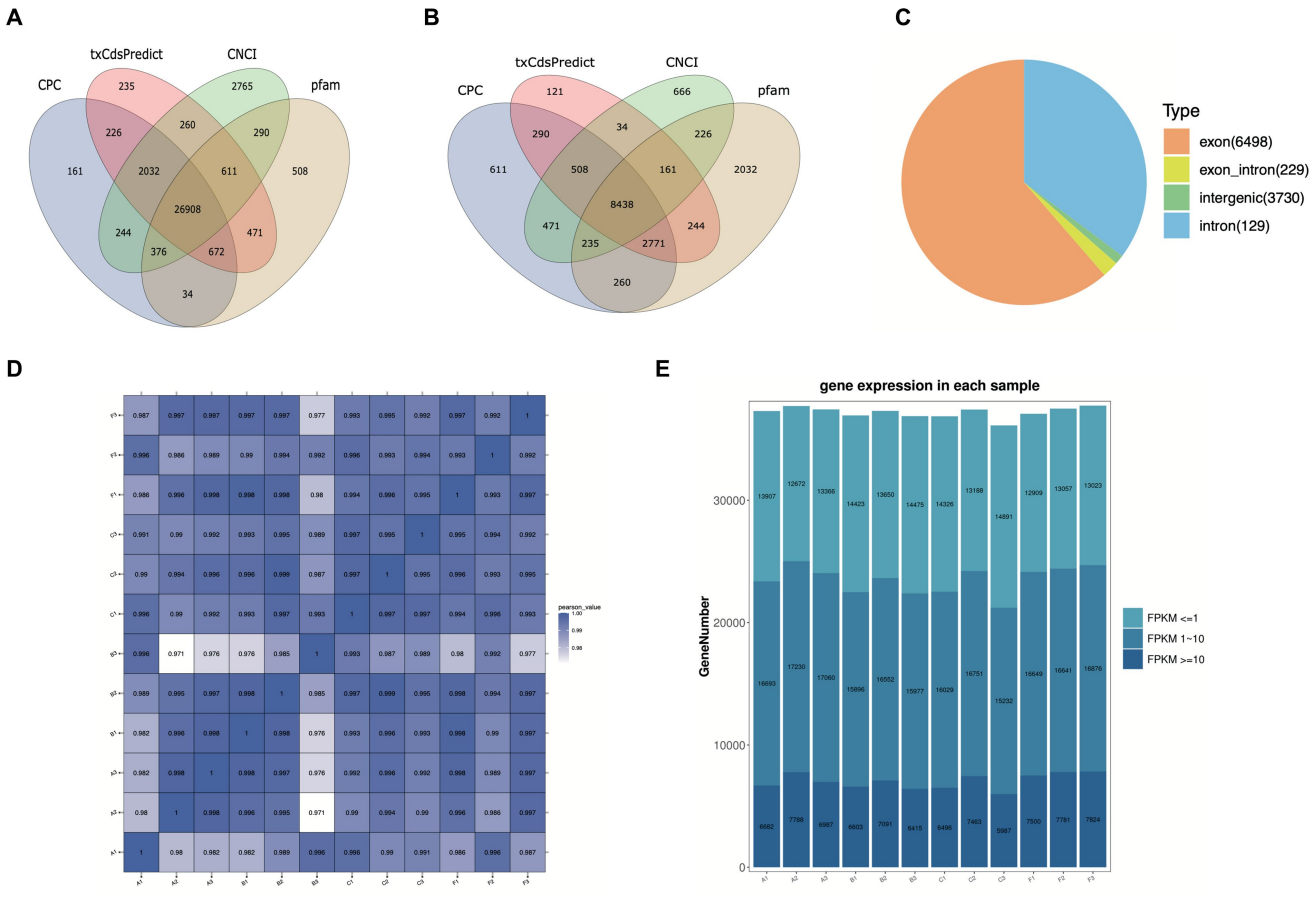
Basic information on twelve WTS libraries in the *R. pedestris* gut under different feeding conditions. The coding capacity predictions of lncRNAs **(A)** and mRNAs **(B)** are shown in Venn diagrams using CNCI, CPC, txCdsPredict, and pfam. **(C)** Summary of circRNA types from twelve libraries. **(D)** The correlation heatmap of all WTS libraries. **(E)** The expression profiles of all genes from twelve WTS libraries are shown by FPKM values. X-axis: twelve WTS libraries, Y-axis: Gene numbers in different libraries.

To explore the lncRNA profiles in the *R. pedestris* gut during different feeding conditions, a total of twelve WTS libraries were constructed and sequenced using the high-throughput MGI2000 platform. Overall, we obtained a total of 1,498,361,064 clean reads from a total of 1,592,518,964 raw data by filtration and quality control, with Q20 ≥ 96% and Q30 ≥ 91% (Supplementary Table S1). Next, clean reads were mapped to the *R. pedestri* genome, indicating the matching rates of each experimental group were more than 77% (Supplementary Table S1). The transcripts were assembled and filtered, and their basic information are shown in Supplementary Figures S1A-C). To identify lncRNAs and mRNAs by coding potential prediction, as shown in Figures 1A,B, 30,599 transcripts were classified as lncRNAs, and 31,139 transcripts were viewed as mRNAs.

On the other hand, circRNAs were also identified and classified from twelve WTS libraries of four experimental treatments. We obtained a total of 1498.36 million clean reads with Q20 ≥ 96.9 and Q30 ≥ 92.66, indicating that they possess high quality after filtering out 1592.5 million raw reads (Supplementary Table S2). Through mapping clean reads to the *R. pedestris* genome, a total of 10,586 circRNAs were identified using CIRI and Find_circ prediction, which were mainly equally distributed in all chromosomes and partial scffolds, while they did not distributed in several scaffolds (data not shown). As evidenced in Supplementary Figure S1D, the length information suggested that the majority of circRNAs were above 3,000 nt. All circRNAs were classified into four types, including 6,498 exon circRNAs, 229 exon_intro circRNAs, 3,730 intergenic circRNAs, and 129 intron circRNAs (Figure 1C). In addition, we predicted the potential function of circRNA coding capacity through cORF (circRNA open reading frame) and IRES (internal ribosome entry site) prediction, suggesting that 5,953 cORFs were identified from 63 to 11,571 bp (Supplementary Figure S1E) and that a total of 8,965 circRNAs possess IRESs from different start and end sites (Supplementary Table S3).

### 3.2 Expression analysis of lncRNAs and circRNAs

**Figure 2:**
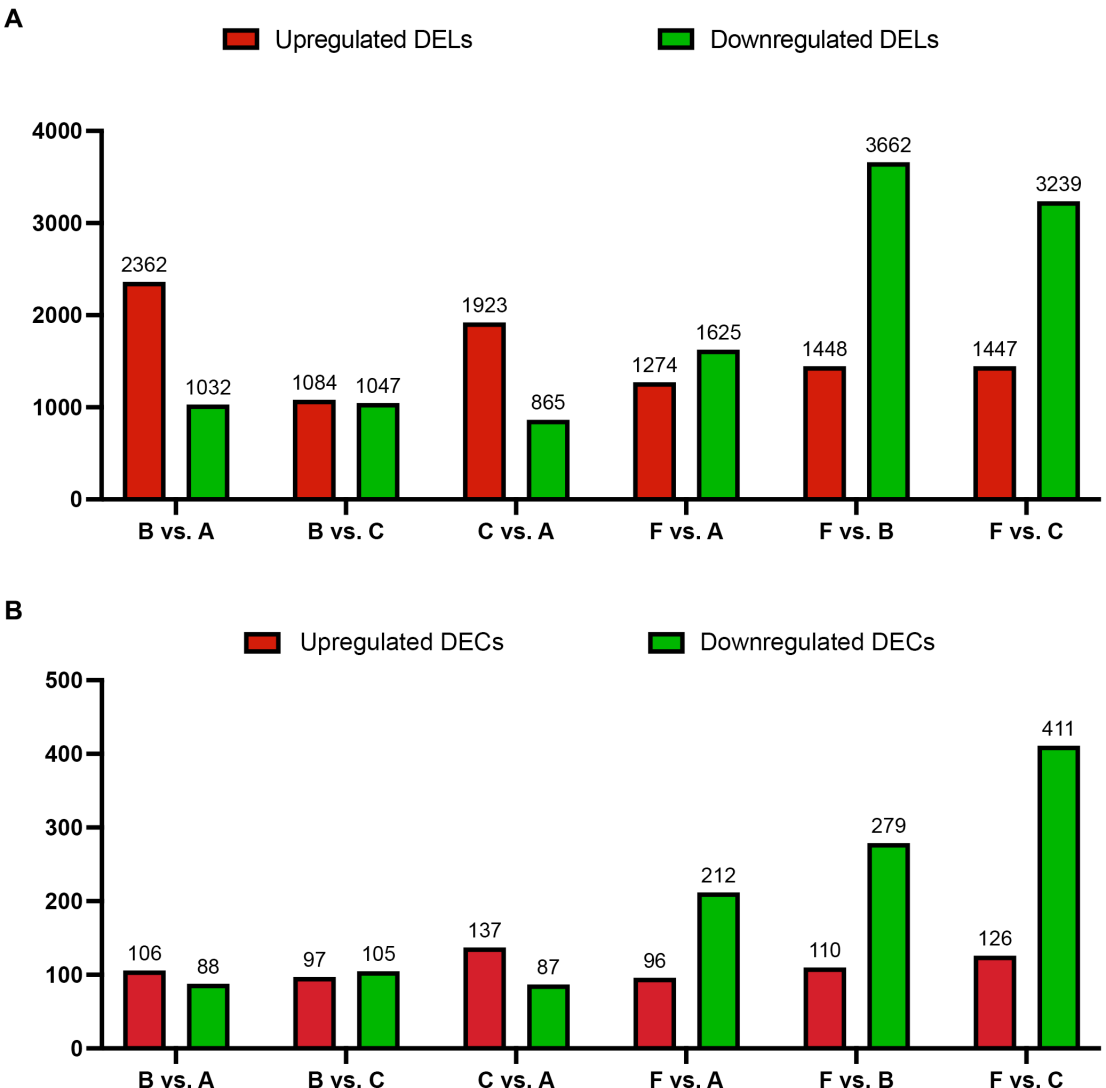
The number of DELs **(A)** and DECs **(B)** for the host genes based on absolute log_2_ |Fold Change| >1 and *p* < 0.001 among the six comparison groups. X-axis: six comparison groups, Y-axis: the number of DELs or DECs for the host genes, respectively. Red and green bars represent upregulated and downregulated DELs or DECs for the host genes, respectively.

In this study, the correlation values exceeded 0.97 among each of twelve WTS libraries, representing the robustness of the biological replicates and the reliability of the sequencing data (Figure 1D), and different numbers of expressed genes were calculated with FPKM values, as shown in Figure 1E. The expression patterns and hierarchical clustering of above genes are shown in a heatmap diagram, and massive clusters are exhibited based on their expression signatures (Supplementary Figure S2). The different numbers of DELs among the six comparison groups is displayed in Figure 2A, suggesting that a total of 9,081 DELs were identified in the antibiotic groups compared to the other groups, including 5,559 upregulated DELs and 3,522 downregulated DELs, and the comparison group of F vs. B observed the largest numbers of DELs. Additionally, a total of 1,854 DECs were selected among the six comparison groups, containing a total number of 672 upregulated DECs and 1,182 downregulated DECs (Figure 2B). The F vs. C group had the majority of DECs, with 126 upregulated DECs and 411 downregulated DECs.

### 3.3 Target prediction and bioinformatic analysis

To further determine the potential regulatory role of lncRNAs, all obtained lncRNAs were applied to predict the potential target mRNAs (lncRNA–mRNA interactions), revealing that a total of 8,850 lncRNA–mRNA pairs, including 3,466 *cis* (nonoverlap)_trans and 5,384 overlap pairs, were calculated in our research. Next, in order to explore the functional annotation of DELs and target genes, we detected different numbers of significantly enriched GO terms among all the comparison groups, containing biological process (BP), cellular component (CC), and molecular function (MF). The GO annotation indicated that the cellular process, cellular anatomical entity, and binding were most enriched (Supplementary Figure S3). The KEGG annotation demonstrated that more than 340 (DELs) and 200 (target genes) KEGG pathways were enriched, and moreover, 20 representative pathways were selected and are shown in Supplementary Figure S4, comprising some immune pathways, such as apoptosis, PI3K-AKT, MAPK-fly, lysosome, Toll and Imd, NOD-like receptor, and TGF-β signaling pathways.

It is well known that circRNAs function as miRNA sponges that regulate target gene expression by binding to miRNAs, so a total of 7,570 circRNAs targeting 2,743 miRNAs were annotated through the interaction prediction. The GO functional annotation of the host genes generated for DECs were also categorized into three types, biological process, cellular component, and molecular function, indicating that DECs were enriched in cellular process, cellular anatomical entity, response to stimulus, catalytic activity, binding, molecular transducer activity, transporter activity, signaling, immune system process, biological regulation, metabolic process, and regulation of biological process (Supplementary Figures S5A–F). For KEGG analysis, the results revealed that the top 20 pathways of DECs distributed in innate immune pathways, metabolism-related pathways, and detoxification-related pathways among the various comparison groups, including apoptosis, lysosome, Toll and Imd, NOD-like receptor, caffeine metabolism, metabolism of xenobiotics by cytochrome P450, nitrogen metabolism signaling pathways, and so forth (Supplementary Figures S5G–L).

Finally, as shown in Table 1, we summarized different numbers of DELs and DECs in innate immune signaling pathways, suggesting that they might be involved in immune activation or regulation, which provide significant implications for the understanding their underlying immune regulation in *R. pedestris*.

**Table 1:** Summary the number of DELs and DECs in immune-related pathways.

Pathway ID	Pathway name	Comparison groups											
		Blank vs. Antibiotic		Blank vs. Control		Control vs. Antibiotic		Field vs. Antibiotic		Field vs. Blank		Field vs. Control	
		DELs	DECs	DELs	DECs	DELs	DECs	DELs	DECs	DELs	DECs	DELs	DECs
ko04624	Toll/Imd signaling pathway	70	2	44	None	58	2	54	2	75	1	86	3
ko04620	Toll-like receptor signaling pathway	31	None	23	None	24	None	23	None	37	None	44	None
ko04621	NOD-like receptor signaling pathway	63	None	55	2	60	2	55	3	88	3	87	2
ko04622	RIG-I-like receptor signaling pathway	50	1	53	None	45	None	52	None	73	1	77	None
ko04630	JAK/STAT signaling pathway	15	1	11	1	16	1	16	1	23	1	21	1
ko04064	NF-κB signaling pathway	35	1	38	None	34	None	37	None	51	1	56	None
ko04151	PI3K-Akt signaling pathway	110	1	81	None	101	None	107	1	140	2	141	3
ko04657	IL-17 signaling pathway	51	1	37	1	40	1	38	2	51	1	48	2
ko04010	MAPK signaling pathway - fly	63	2	45	None	72	None	60	None	105	1	105	2
ko04142	Lysosome	169	5	151	5	145	7	212	10	254	9	243	13
ko04668	TNF signaling pathway	45	None	33	None	37	None	39	None	55	None	60	None
ko04350	TGF-β signaling pathway	29	None	20	None	29	None	40	None	58	1	46	1
ko04214	Apoptosis-fly	29	None	18	None	23	None	40	None	57	None	51	None
ko04625	C-type lectin receptor signaling pathway	33	1	30	None	28	None	32	None	51	1	52	None

### 3.4 Identification and characterization of *defensin* and *lysozyme* family genes

**Figure 3:**
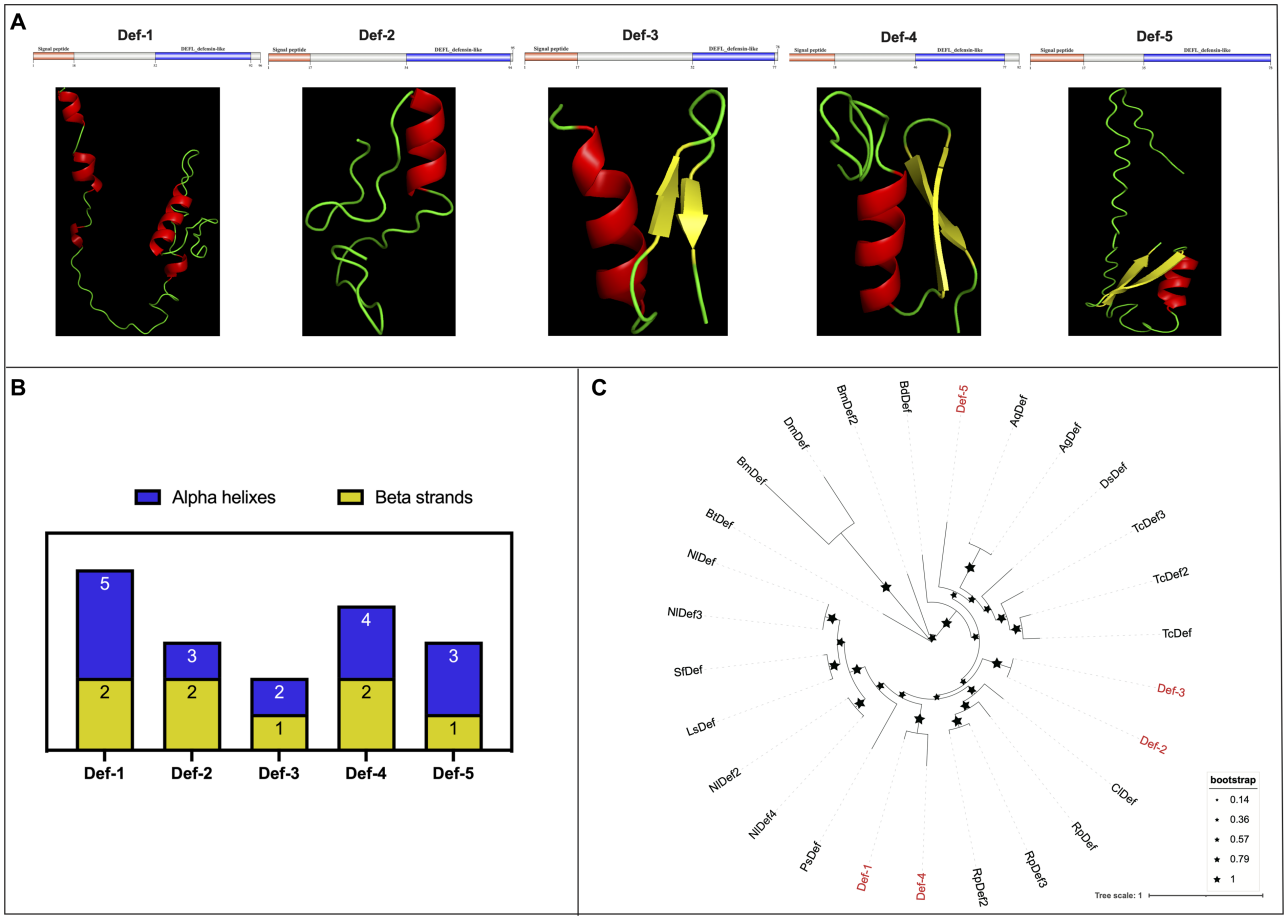
Protein structures and phylogenetic analysis of defensins. **(A)** Schematic and tertiary structures of five defensin proteins of *R. pedestris*. 3D models and schematic representation of the domain topology of all defensins were generated and predicted using SWISSMODEL and SMART, indicating alpha-helix (red), beta-fold (green), and random coil (yellow). SP: signal peptide. **(B)** Summary of the number of alpha helixes and beta strands of five defensin proteins in *R. pedestris.* **(C)** Phylogenetic relationships of *defensins* from *R. pedestris* and other insects. The phylogenetic tree was built using MEGA 9.0 and 1,000 bootstrap support replicates. The value bar represents the genetic distance, and only branch support values for the main branches are shown by black pentacles with proportional sizes. Five *R. pedestris defensins* are indicated in red, and the GenBank accession numbers used for phylogenetic tree construction are found in Supporting Information: Supplementary Table S4.

In the present research, we identified a total of five *defensins* from WTS data in the *R. pedestris* gut, namely, *Def-1*, *Def-2*, *Def-3*, *Def-4*, and *Def-5*, and of them, *Def-5* was reported in a previous study (Futahashi et al., 2013). The characteristics of cDNA and protein sequences, such as sequence length, molecular weight, theoretical isoelectric point, domain locations, and NCBI accession numbers, are summarized in Table 2. In detail, the cDNA sequences of *defensins* varied from 291 bp (*Def-1*) to 222 bp (*Def-5*), encoding 96 amino acids (aa) to 73 aa with MWs from 10.62 to 5.87, respectively. Furthermore, five *R. pedestris* defensins were observed to possess a 17 or 18 aa signal peptide and a typical defensin-like (DEFL) domain (nearly 30 aa) that has six conserved cysteines for forming intrachain disulfide bonds (Supplementary Figure S6A), with positive or negative hydropathicity. Reasonable 3D models of all *R. pedestris* defensins were predicted and selected, revealing that their structures contain different numbers of alpha helixes and beta strands (Figures 3A,B). Here, a phylogenetic tree was performed to validate the orthologous relationships of *defensins* between *R. pedestris* and other insects, suggesting that *Def-2* and *Def-3*, as well as *Def-1* and *Def-4,* were clustered in the same branch, and *Def-5* grouped with the corresponding *defensins* of *Anopheles gambiae* and *A. quadriannulatus* (Figure 3C).

**Table 2:** The characterization of cDNA and protein sequences of *defensin* and *lysozyme* family genes.

Gene name	ORF length (bp)	Amino acids length (aa)	Molecular weight (kDa)	Theoretical pI	Grand average of hydropathicity (GRAVY)	Signal peptide motif (aa)	Functional domain (aa)	NCBI accession number	Reference
Def-1	291	96	10.62	8.70	−0.057	1–18	DEFL_defensin-like: 52–92	OR026580	This study
Def-2	288	95	10.14	6.26	0.186	1–17	DEFL_defensin-like: 54–94	OR026581	This study
Def-3	237	78	8.28	6.38	0.371	1–17	DEFL_defensin-like: 52–77	OR026582	This study
Def-4	249	82	9.41	9.00	−0.191	1–18	DEFL_defensin-like: 46–77	OR026583	This study
Def-5	222	73	5.87	8.34	−0.327	1–17	DEFL_defensin-like: 35–73	BAN20110	Futahashi et al., 2013
LyzI-1	507	168	18.86	4.73	−0.251	1–26	Destabilase: 39–154	OR026584	This study
LyzI-2	459	152	16.92	5.33	−0.292	1–20	Destabilase: 29–146	OR026585	This study
LyzI-3	516	171	18.95	6.13	−0.328	1–29	Destabilase: 39–154	OR026586	This study
LyzI-4	534	177	19.47	4.88	−0.040	1–21	Destabilase: 39–154	OR026587	This study
LyzC-1	468	155	17.49	8.43	−0.474	1–20	LYZC_Invert: 23–140	OR026588	This study
LyzC-2	639	212	23.75	4.81	−0.275	1–14	LYZC_Invert: 15–138	OR026589	This study
LyzC-3	534	177	20.11	9.32	−0.209	None	Transmembrane region: 35–54 LYZC_Invert: 57–176	BAN20103	Futahashi et al., 2013
LyzC-4	483	160	17.86	7.46	−0.150	None	LYZC_Invert: 44–159	BAN20138	Futahashi et al., 2013
LyzB-1	669	222	25.04	6.23	−0.319	1–17	Glyco_25: 23–189	OR026590	This study
LyzB-2	675	224	25.05	9.30	−0.447	1–18	Glyco_25: 24–188	OR026591	This study
LyzB-3	666	221	24.96	9.32	−0.193	1–17	Glyco_25: 23–187	OR026592	This study

**Figure 4:**
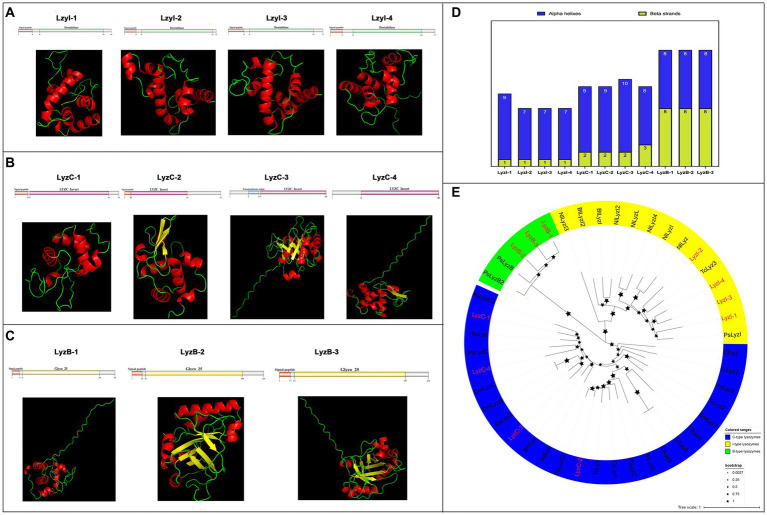
Protein structures and phylogenetic analysis of lysozymes. Schematic and tertiary structures of four I-type lysozymes **(A)**, C-type lysozymes **(B)**, and B-type lysozymes **(C)** of *R. pedestris*. 3D models and schematic representation of the domain topology of all lysozymes were generated and predicted using SWISSMODEL and SMART, indicating alpha-helix (red), beta-fold (green) and random coil (yellow). SP, Signal peptide; TM, Transmembrane region. **(D)** Summary of the number of alpha helixes and beta strands of lysozyme proteins in *R. pedestris.* **(E)** Phylogenetic relationships of *lysozymes* from *R. pedestris* and other insects. The phylogenetic tree was built using MEGA 9.0 and 1,000 bootstrap support replicates. The value bar represents the genetic distance, and only branch support values for the main branches are shown by black pentacles with proportional sizes. All different types of *R. pedestris lysozymes* are indicated in red, and GenBank accession numbers used for phylogenetic tree construction are found in Supporting Information: Supplementary Table S4. I-type lysozymes, C-type lysozymes, and B-type lysozymes of selected insects are highlighted in yellow, blue and green, respectively.

Next, a total of eleven *lysozyme* family genes were selected and characterized in *R. pedestris*, which were classified into three subfamilies, I-type (*LyzI-1* to *−4*), C-type (*LyzC-1* to *−4*) and B-type (*LyzB-1* to *−3*), of which, *LyzC-3* and *LyzC-4* have been reported in Futahashi et al., 2013. Concretely, the cDNA length of all *lysozyme*s ranged from 675 bp (*LyzB-2*) to 459 bp (*LyzI-2*), and their protein length ranged from 224 aa to 152 aa with negative hydropathicity (Table 2). Among these lysozymes, the pI values ranged from 9.32 (LyzB-3) to 4.73 (LyzI-1), and LyzB-2 (25.05) and LyzI-2 (16.92) possess the largest and smallest molecular weights, respectively. The results of domain prediction showed that the I-type lysozymes have a signal peptide motif and Destabilase domain, which contains ten conserved cysteines that form disulfide bonds (Figures 4A and Supplementary Figure S6B). As shown in Figures 4B and Supplementary Figure S6C, the C-type lysozymes shared a typical invertebrate LYZ_C_invert domain, which has seven conserved cysteines to form disulfide bonds, while a signal peptide or transmembrane region existed in three C-type lysozyme members of *R. pedestris*. The signal peptide and Glyco_hydro_25 (GH 25) domain consisted of all B-type lysozymes (Figures 4C and Supplementary Figure S6D). Moreover, the 3D protein structures of eleven lysozymes were constructed with the online programs (Figures 4A–C), showing that each B-type lysozyme has eight alpha helixes and eight beta strands in *R. pedestris*, composed of eight β-strands and six α-helices in the Glyco_hydro_25 (GH 25) domain. LyzI-2, LyzI-3 and LyzI-4 consist of seven alpha helixes and one beta strand, while LyzI-1 contains more alpha helixes than other I-type lysozymes and one beta strand in *R. pedestris* because the Destabilase domain of I-type lysozyme is commonly characterized by six α-helices and one β-strand. The total number of alpha helixes and beta strands of *R. pedestris* LyzC-3 was than in other C-type lysozymes (Figure 4D). Finally, a phylogenetic tree was constructed using the protein sequences of three lysozyme subtypes from *R. pedestris* and other insects using the NJ method and 1,000 bootstrap replicates, supporting that all selected *lysozymes* were grouped together in each well-supported clade along with three distinct subfamilies, as our classification, with higher branch support values (Figure 4E).

### 3.5 Expression and correlated analyses of *defensin*s and *lysozymes*

**Figure 5:**
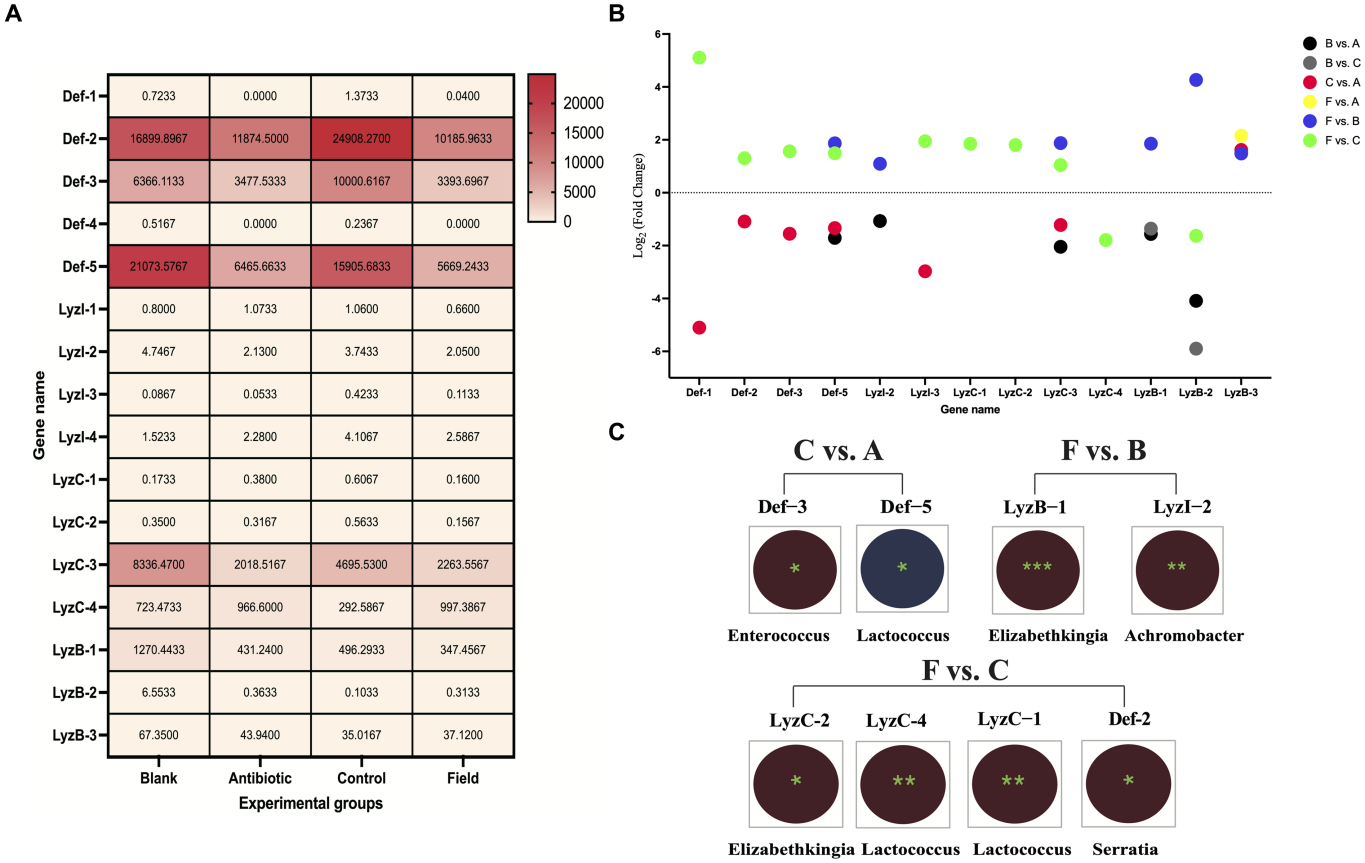
Expression and correlated analyses of *defensins* and *lysozymes*. **(A)** Expression heatmap of *defensins* and *lysozymes* under four rearing conditions in *R. pedestris* guts. All grids, with different colors corresponded to different expression levels, shown by FPKM values as mean (*N* = 3). **(B)** Expression profiles of DE *defensin* and *lysozyme* family genes among the six comparison groups. X-axis: the name of DE AMP genes, Y-axis: Log_2_ (Fold Change) values of all above DE AMP genes. Different circle colors represent different comparison groups, including B vs. A, B vs. C, C vs. A, F vs. A, F vs. B, and F vs. C. **(C)** Integrated analyses of gut bacterial changes and two DE AMP genes from C vs. A, F vs. B, and F vs. C groups with Pearson correlation coefficients using R software (version 2.15.3) in the *R. pedestri* guts under different rearing conditions. Red and blue colors represent negative and positive correlation, respectively, and the correlation significance is shown by ^***^ (*p* < 0.001), ^**^ (*p* < 0.01), and * (*p* < 0.05).

First, overall expression patterns of *defensins* and *lysozymes* showed that *Def-2*, *Def-3*, *Def-5*, *LyzC-3*, *LyzC-4*, and *LyzB-1* exhibited relatively high distributions (FPKM values more than 100) among all the experimental groups (Figure 5A). On the contrary, *Def-4*, *LyzI-3*, *LyzC-1*, and *LyzC-2* were relatively low expression (FPKM values less than 1) among all the treatment groups. Of note, the expression levels of *Def-1* and *Def-4* were not detected in Antibiotic group. To investigate the differentially expressed patterns of *defensin* and *lysozyme* family genes in the *R. pedestris* gut under different rearing conditions, bar charts were generated using log_2_ (fold change) values based on WTS data (Figure 5B). Concretely, a total number of 32 DE *defensins* and 30 DE *lysozymes* were identified from the Field group compared to the Control and Blank groups. Only one AMP, *LyzB-3*, differentially expressed in the F vs. A group. In addition, there were different numbers of DE *defensins* and *lysozyme*s in the B vs. A and C vs. A groups, but we only selected DE *LyzB-1* and *LyzB-2* without DE *defensins* in the B vs. C group.

The Pearson correlation coefficients were estimated to investigate the correlation of above DE AMPs and most changes in the gut bacterial abundance at the genus level among all the comparison groups according to WTS and 16S rRNA sequencing data, which could provide a good and reliable model to elucidate their associations in *R. pedestris*. As shown in Figure 5C, in the results of the F vs. C group, we found maximal significantly correlated pairs, including three DE *lysozymes*, one DE *defensin*, and three genera in gut bacteria. In the C vs. A group, two DE *defensins* (*Def-3* and *Def-5*) were significantly negatively and positively associated with two genera in gut bacterial changes (*Enterococcus* and *Lactococcus*), respectively, while significantly associated pairs of *lysozyme*-gut bacteria were not observed. Conversely, only two DE *lysozymes* (*LyzB-1* and *LyzI-2*) were significantly negatively associated with changes in *Elizabethkingia* and *Achromobacter* genera, respectively.

Finally, a total of three DE AMPs, four DELs and two DECs were selected for qRT–PCR validation, and the results showed that their expression trends were consistent with WTS data (Supplementary Figure S7).

### 3.6 The potential ncRNA-mediated networks of *defensins* and *lysozymes*

**Figure 6:**
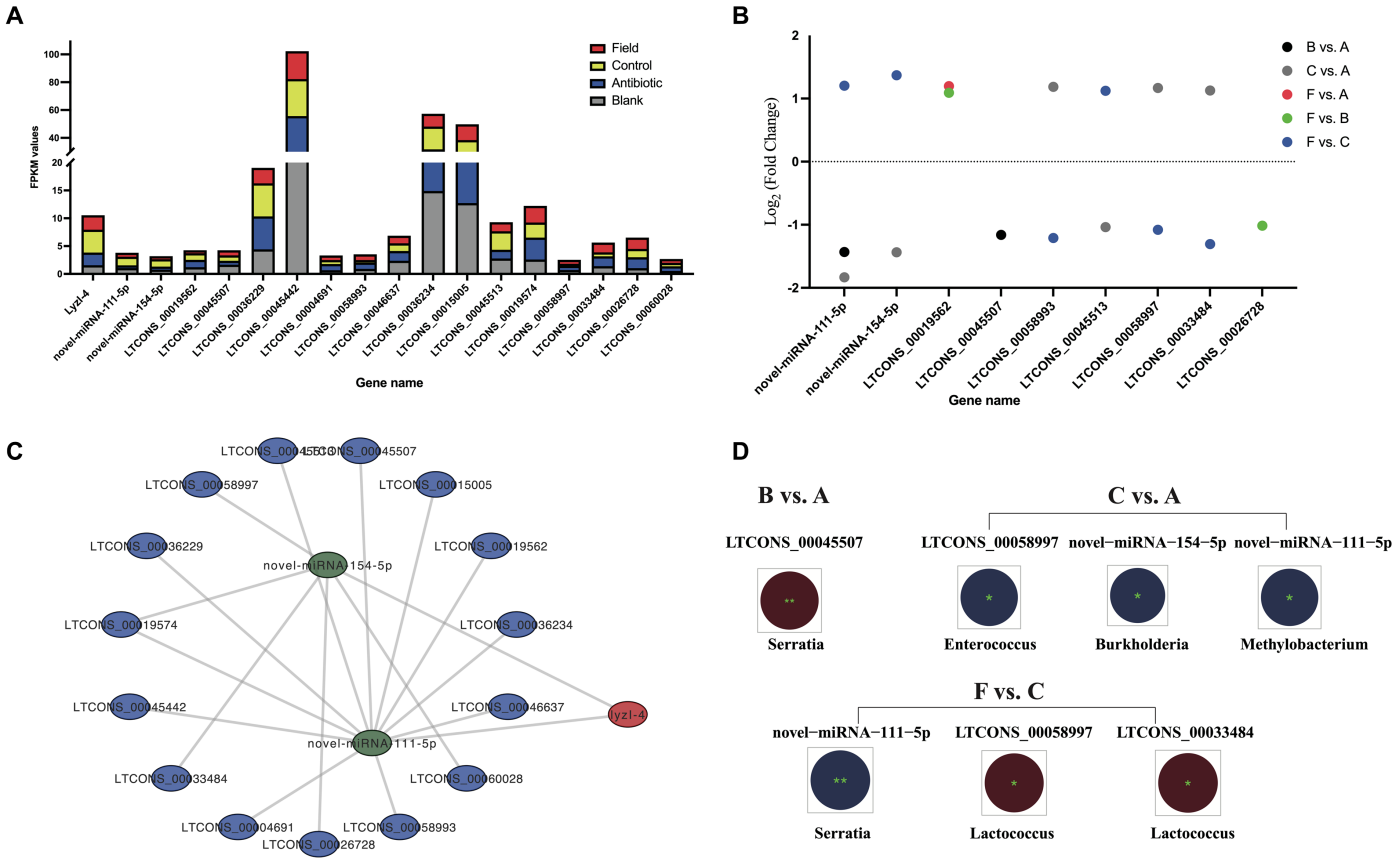
The ceRNA network of lncRNA–miRNA–lyzI-4. **(A)** The expression levels of the ceRNA network of all lncRNAs, miRNAs and lyzI-4 among four experimental groups. X-axis: the ceRNA network of all lncRNA, miRNA and lyzI-4, Y-axis: FPKM values. **(B)** Differential expression of lncRNAs and miRNAs for ceRNA construction among the six comparison groups. X-axis indicates the name of DELs and DECs, and Y-axis indicates Log_2_ (Fold Change) values of corresponding DELs and DECs. Different circle colors represent the different comparison groups, including B vs. A, C vs. A, F vs. A, F vs. B, and F vs. C. **(C)** The ceRNA networks of *lyzI-4* based on WTS annotation and target prediction by Cytoscape software (version 3.9.1). The red node indicates *lyzI-4*, the green nodes indicate miRNAs, and the blue nodes indicate lncRNAs. **(D)** Integrated analysis of gut bacterial changes and above DELs and DECs from the B vs. A, C vs. A, and F vs. C groups with Pearson correlation coefficients using R software (version 2.15.3) in the *R. pedestri* gut samples under different rearing conditions. Red and blue colors represent the negative and positive correlation, respectively, and the correlation significance is shown by ^**^ (*p* < 0.01) and ^*^ (*p* < 0.05).

Based on the negatively regulatory mechanism between miRNAs and corresponding target genes, a total number of twenty-nine miRNA–*defensin* and seventy-four miRNA–*lysozyme* pairs were predicted and selected to construct a interaction network, containing all *defensin* and *lysozyme* genes in *R. pedestris* (Supplementary Figure S8A). Subsequently, a ceRNA network of lncRNA–miRNA–LyzI-4 pairs were found (Figure 6C and Supplementary Table S6). In brief, there were fifteen lncRNAs and two miRNAs in this network, and LTCONS_00019574 target to both novel-miRNA-154-5p and novel-miRNA-111-5p for indirectly regulating the *LyzI-4* expression. The basic information of above lncRNAs is summarized in Table 3, showing that they ranged from 12,896 nt (LTCONS_00033484) to 1,092 nt (LTCONS_00026728) in length and distributed on different chromosomes of *R. pedestris*, and some of them were classified into cis_overlap and cis_down20K. Besides, the precursor and mature sequences of novel-miRNA-154-5p and novel-miRNA-111-5p are both 56 nt and 22 nt in length, respectively, exhibiting typical stable stem-loop secondary structures (Supplementary Figure S8B).

**Table 3:** The basic information of lncRNAs for ceRNA construction.

Name	Gene symbol	Length (nt)	Classification	Chromosome
LTCONS_00019562	LXLOC_011657	12,813	cis _overlap	Chr2
LTCONS_00019574	LXLOC_011653	6,956	cis _overlap	Chr2
LTCONS_00045507	LXLOC_026951	1,511	cis _overlap	Chr4
LTCONS_00036229	LXLOC_021436	5,227	None	Chr3
LTCONS_00045442	LXLOC_026925	1979	None	Chr4
LTCONS_00004691	LXLOC_002787	10,523	None	Chr1
LTCONS_00058993	LXLOC_034898	7,613	cis _overlap	ChrX
LTCONS_00046637	LXLOC_027572	1,461	None	Chr5
LTCONS_00036234	LXLOC_021435	6,941	None	Chr3
LTCONS_00015005	LXLOC_008835	6,239	None	Chr1
LTCONS_00045513	LXLOC_026954	7,528	cis _overlap	Chr4
LTCONS_00058997	LXLOC_034900	5,574	cis _overlap	ChrX
LTCONS_00033484	LXLOC_019780	12,896	cis _down20K	Chr3
LTCONS_00026728	LXLOC_015869	1,092	cis _down20K	Chr2
LTCONS_00060028	LXLOC_035533	2,751	None	ChrX

Overall, as shown in Figure 6A, the expression changes of above lncRNAs and *LyzI-4* among all the experimental groups displayed the varying trends were observed to higher than the expression profiles of novel-miRNA-154-5p and novel-miRNA-111-5p. Of these lncRNAs and miRNAs, a total of seven DELs and two DE miRNAs (DEMs) were identified in the *LyzI-4*-related ceRNA network under feeding environmental changes of *R. pedestris* (Figure 6B). We further evaluated the correlated relationships between above DELs and DEMs, as well as most changes in the gut bacterial abundance at the genus level among all the comparison groups. Concretely, the results illustrated that DE LTCONS_00045507 was significantly negatively correlated with *Serratia* genus variation in the B vs. A group. Conversely, DE novel-miRNA-154-5p, novel-miRNA-111-5p and LTCONS_00058997 were significantly positively associated with changes in *Burkholderia*, *Methylobacterium* and *Enterococcus* genera in the C vs. A group, respectively Ultimately, the alterations in *Serratia* and *Lactococcus* genera significantly positively and negatively influenced the expression of novel-miRNA-111-5p, as well as LTCONS_00058997 and LTCONS_00033484 in the F vs. C group, respectively.

## 4 Discussion

*Riptortus pedestris* is a widely distributed pest in East Asia and result in significant yield losses to soybeans by sucking pods, so it is urgent to explore a model of the plant–insect–microbe interactions for pest management (Huang et al., 2021; Wei et al., 2023). With the rapid development of high-throughput sequencing, a deep understanding and elucidation of the biological function of ncRNAs, such as lncRNAs, circRNAs and miRNAs, which contribute to a new aspect of the adaptive evolution in insects, are rapidly improving (Hu et al., 2018; Zhang et al., 2021, 2023; Zhou et al., 2021a, 2022b). In previous reports, some AMPs were found to exert functions in distinguishing gut commensal symbionts from pathogens in *R. pedestris* (Kim et al., 2015; Lee et al., 2017), and a subsequent study found that its gut symbionts play complementary roles under AMP deletion (Lee et al., 2022). Here, the current study is the first to demonstrate the large-scale identification and characterization of ncRNAs and their potential interaction with *R. pedestris* AMPs, and the associations of gut bacterial changes at the genus level and above DE ncRNAs and AMPs under feeding environmental changes. Our work aimed to pave the way for pest control and adaptive evolution in hemipterous pests.

The lncRNA and circRNA profiles using WTS technology provide deeper insight into their regulatory mechanism under feeding environmental changes in the *R. pedestris* gut. First, in order to select massive lncRNAs, all clean reads from each treatment group were independently mapped to the *R. pedestris* reference genome, exceeding 77% mapping ratio, and moreover, the transcripts were filtered out, yielding a total of 30,599 lncRNAs and 31,139 mRNAs by protein-coding potential prediction. Meanwhile, we further obtained a total of 10,586 circRNAs, which were divided into exon circRNAs, exon_intron circRNAs, intergenic circRNAs, and intron circRNAs, and their lengths were nearly above 3,000 nt. In this study, the numbers of lncRNAs and circRNAs were more than those in other insects, but the types of these circRNAs are similar to those in model insect (Wu et al., 2016; Hu et al., 2018; Zhang J. et al., 2020; Zhang S. et al., 2020; Yin et al., 2021; Zhang et al., 2021). Furthermore, we identified abundant DELs and DECs among the six comparison groups, consisting of a total number of 5,559 upregulated DELs and 3,522 downregulated DELs as well as a total number of 339 upregulated DECs and 387 downregulated DECs in comparison between antibiotic and other treatments. Our results are in agreement with these previous findings that different numbers of DE ncRNAs were calculated from insect tissues or cells under various treatments, such as *B. mori*, *Tribolium castaneum* and *Aedes aegypti* (Etebari et al., 2016; Hu et al., 2018; Yin et al., 2021; Abd El Halim and Ali, 2022).

For target prediction and functional annotation of lncRNAs and circRNAs, 8,850 lncRNA–mRNA pairs, including 3,466 *cis* (nonoverlap) trans and 5,384 overlap pairs, and 7,570 circRNAs targeting 2,743 miRNAs were predicted in this study. Although there is much information on the lncRNAs or circRNAs involved in the regulation of innate immune responses in model insects, little is known about the mode of them in nonmodel insects. For this objective, in the present study, GO and KEGG enrichment analyses were performed to annotate the function of above DELs, DECs, and their targets, suggesting that they were mainly involved in numerous immune signaling pathways, such as the Toll and Imd, NOD-like receptor, PI3K-AKT, and MAPK-fly signaling pathways. It has been revealed that insects depend on humoral immunity against pathogens, such as Toll, Imd, and JAK–STAT, which are the most important signaling cascades for the immune response and metabolism (Yang and Hultmark, 2017). In keeping with our data, GO and KEGG analyses were also used to explore the biological functions and potential mechanisms of circRNAs in BmCPV-infected *B. mori* midguts and fat bodies, important metabolic and immune organs in maintaining normal physiological functions, suggesting that abundant immune circRNAs were identified in response to BmCPV infection using circRNA sequencing (Hu et al., 2018; Yin et al., 2021). In addition, a total of 7,448 DELs and 12,263 lncRNA–mRNA pairs were predicted and obtained based on *cis*-regulation analysis in the normal and BmNPV-infected BmN cells, some of which contributed to immune and metabolic processes according to GO and KEGG enrichment analyses (Zhang S. et al., 2020). The red flour beetle, *T. castaneum*, was also found to be a useful model for several biological research areas because it is one of the major pests of stored agricultural products, generating numerous DELs and target genes potentially related to immune priming under bacterial infection (Abd El Halim and Ali, 2022). Notably, in this research, as DECs were not annotated in some innate immune pathways, it is possible that circRNAs cannot involve in the regulation of these pathways in response to environmental variations in the *R. pedestris* gut. Overall, all these data on differential expression, target prediction, and functional annotation of lncRNAs and circRNAs in the *R. pedestris* gut could provide new evidence on insect adaption to environmental changes by post-transcriptional regulation.

Studies on insect AMPs, known as the ‘backbone’ of the innate immune system, have attracted our attention, and to date, there are 98 types of AMPs from insect cells or tissues under different developmental phases, such as *Hyalophora cecropia*, *D. melanogaster*, *A. gambiae*, *T. castaneum* and *Apis mellifera* (Azmiera et al., 2022). In general, AMPs are small amino acid sequences that are positively charged (cationic) due to the prevalence of basic amino acids, such as arginine, lysine, and histidine, and they are classified into four subtypes: α-helical peptides (cecropin and moricin), cysteine-rich peptides (insect defensin and drosomycin), proline-rich peptides (apidaecin, drosocin, and lebocin), and glycinerich peptides/proteins (attacin and gloverin) (Yi et al., 2014). For their functional investigations in insects, AMPs have been validated to possess broad antimicrobial activity against gram-positive and gram-negative bacteria, viruses, fungi, and parasites (Mahlapuu et al., 2016). For instance, many of them produce a bactericidal effect by directly destroying the microbial membrane, penetrating the cytoplasm, and finally interacting with intracellular targets such as DNA, RNA or ribosomal synthesis, or they could involve in selective immunomodulatory effects, including cell proliferation and the production of proinflammatory cytokines (Azmiera et al., 2022). In this study, we selected five *defensins* for the first time, of which *Def-5* was identified from a previous report (Futahashi et al., 2013), and we observed that *Def-1* was close to *Def-4*, and *Def-2* was clustered with *Def-3* based on phylogenetic results. In hemipterous insects, a limited number of *defensins* has been found in *Chlorochroa ligata*, *Euschistus conspersus*, and *Thyanta pallidovirens* (Marshall et al., 2023). The results of protein sequence prediction of five *R. pedestris* defensins showed that their protein length ranged from 96 to 73 aa with signal peptides and the typical characteristics of both defensin and AMPs, such as DEFL domain, six-cysteine motifs, and disulfide bridges (Li Y. et al., 2021). Likewise, the sequence and functional observations of defensins in *Cimex lectularius* and *Rhodnius prolixus* have suggested that defensins share DEFL domains with conventional cysteine residues and antimicrobial or antiviral effects (Kaushal et al., 2016; Li Y. et al., 2021). On the other hand, *lysozymes*, the glycoside hydrolase (GH) family members, play important roles in protective immunity against invasion by bacteria, fungi and viruses, containing the C-type (chicken-type, GH22), G-type (goose-type, GH23), I-type (invertebrate-type, GH22), V-type (viral type, GH24) and B-type (GH25) (Feng et al., 2020). Since the first insect *lysozyme* was found in honey bees, more than fifty *lysozyme* genes have been identified from different insects (Mohamed et al., 2016), but little is known about the *R. pedestris lysozymes*. A total of eleven *lysozymes* in *R. pedestris*, more than those in some hemipterous pests such as *Nezara viridula*, *Chlorochroa ligata*, *Plautia stali,* and *Thyanta pallidovirens*, were identified and characterized (Marshall et al., 2023), and among these *lysozymes*, three subtypes were classified based on domain prediction and phylogenetic construction. They possess the domain of glycoside hydrolase that is shared in different lysozymes to perform lytic action and muramidase activity on the cell wall peptidoglycan (PGN) of bacteria (Feng et al., 2020). Similarly, three subtypes of six *lysozymes* in the brown-winged green stinkbug *Plautia stali*, were obtained (two *LyzB*, three *LyzC*, and one *LyzI*) from transcriptome libraries, some of which contributed to the response to *Escherichia coli* and gram-positive *Micrococcus luteus* stimulation and were controlled by Toll and Imd pathways (Nishide et al., 2019). Taken together, all these data provides a new basis for future investigations on *defensins* and *lysozymes* involving in adaptive regulation of hemipterous insects.

Although there are accumulating reports on lncRNAs and circRNAs that involved in controlling the transcription of immune genes by interaction with various signaling factors in insects (Zhou et al., 2021a,b; Yin et al., 2021; Zhou et al., 2022a,b; Lin et al., 2023), there is little evidence on the ncRNA-mediated networks of AMPs in *R. pedestris* under environmental changes. In the current study, we obtained the different number of ncRNAs to explore the regulatory networks of two AMPs in *R. pedestris* at post-transcriptional level, combined with our previous work that reported miRNA profiles of bean bugs in response to feeding conditional changes (Ren et al., 2023). First, we construct a interaction network of miRNA–*defensin*/*lysozyme* in *R. pedestris* by target prediction, including twenty-nine miRNA–*defensin* and seventy-four miRNA–*lysozyme* pairs. Furthermore, we observed that a *lysozyme* gene were regulated by a lncRNA-mediated ceRNA network, while the ceRNA network of circRNA–miRNA–*lysozyme* and circRNA/lncRNA–miRNA–*defensin* was not found. Hence, the presence of above interactions and ceRNA network in the *R. pedestris* gut of our study could be the result of the regulation for feeding environmental changes.

To the best of our knowledge, the gut microbiota is a complex and dynamic biological system composed of commensal, symbiotic and pathogenic microbial communities, including bacteria, archaea, fungi, protozoa, and viruses. It has been suggested that changes or disturbances in the gut microbial community can affect the immune function and environmental adaption in animals, for instance, by influencing the expression of innate immune genes or effectors (Evariste et al., 2019). Based on these observations, we found various DE *defensins* and *lysozymes* under rearing conditional changes in the *R. pedestris* gut, including four *defensin* members and nine *lysozyme* members, and moreover, seven lncRNAs and two miRNAs in the *LyzI-4*-related ceRNA network were shown to differential expression by antibiotic treatment and different feeding treatments. In agreement with our investigations, an imbalance in gut microbial homeostasis affected AMP expression in *B. mori* under *Enterobacter cloacae* infection by acetamiprid feeding (Li F. C. et al., 2021). For the fall armyworm (FAW), *Spodoptera frugiperda*, diets and different survival conditions impacted its gut microbiota and immune system, indicating significant changes in *lysozymes*, *attacins* and *cecropins* genes, and gut microbiome communities between captive and wild samples (Zheng et al., 2023). Duplouy et al. (2020) demonstrated that alterations in the gut microbiota involved in the upregulation of an antimicrobial peptide, *attacin*, but it did not affect life history traits or metabolism in Glanville fritillary butterflies (*Melitaea cinxia*, L.) using antibiotic treatment. Similarly, some gut microorganisms of *Hyphantria cunea* can stimulate host immune systems, such as several antimicrobial peptides (particularly *gloverin 1*), thereby contributing to resistance to entomopathogens and nucleopolyhedrovirus (NPV) using normal and germ-free larvae (Li et al., 2023). Next, we try to construct the associations between shifts in the gut bacteria and above DE AMPs and ncRNAs in *R. pedestris*. In previous reports, *R. pedestris* holds a bacterial symbiont belonging to the genus *Burkholderia* in midgut crypts from the field soils at every generation (Kikuchi et al., 2007; Takeshita and Kikuchi, 2017), and this symbiont could provide a strong fitness benefit to the stinkbugs in environments with high insecticide treatment (Kaltenpoth and Flórez, 2020). Our data illustrated that the different numbers of genera in gut bacterial changes were significantly positively or negatively associated with some DE *defensins*, and *lysozymes* and related DE ncRNAs among the six comparison groups in the whole gut of *R. pedestris* using the integrative analysis of WTS and 16S rRNA sequencing with Spearman correlation analysis. On the basis of the above data, these findings favor our initial expectations that changes in AMP expression through the ncRNA-involved axis in concert with given gut bacteria could be the consequence of adapted environmental shifts in *R. pedestris*, which provided more evidence on the regulatory mechanisms of innate immunity in *R. pedestris* at post-transcriptional level under different environments.

## 5 Conclusion

In conclusion, WTS technology was executed to investigate lncRNA and circRNA profiles in the *R. pedestris* gut under different feeding conditions. In total, we obtained abundant lncRNAs and circRNAs, including different numbers of DELs and DECs among the six comparison groups, by quality control and bioinformatics analysis. Functional enrichments showed that they played important roles in innate immune pathways of *R. pedestris*. Later, we identified and characterized five *defensin* and eleven *lysozyme* family genes in *R. pedestris*, and phylogenetic analysis revealed their classification and evolutionary conservation in insects. One miRNA target network of *defensins* and *lysozymes* and a ceRNA network of one *lysozyme* were predicted and built in *R. pedestris*, which laid the foundation for further studies to validate these findings by experimental verification. Finally, different numbers of *defensins*, and *lysozymes* and related ncRNAs were differentially expressed among the various comparison groups, and besides, they were significantly negatively or positively associated with gut bacterial changes at the genus level in *R. pedestris* in response to different feeding conditions. Overall, these observations can provide a basis for adaptive evolution and pest management of *R. pedestris*.

## Data availability statement

The datasets presented in this study can be found in online repositories. The names of the repository/repositories and accession number(s) can be found at: https://www.ncbi.nlm.nih.gov/genbank/, PRJNA638739, OR026580 to OR026592.

## Ethics statement

The animal study was approved by Animal Care and Use Committee of Nankai University. The study was conducted in accordance with the local legislation and institutional requirements.

## Author contributions

YR: Writing – review & editing, Writing – original draft, Visualization, Supervision, Methodology, Investigation, Formal analysis, Conceptualization. SF: Writing – review & editing, Validation, Investigation, Formal analysis, Data curation. WD: Writing – review & editing, Visualization, Methodology, Investigation, Formal analysis. JC: Writing – review & editing, Software, Resources, Methodology, Investigation, Data curation. HX: Writing – review & editing, Supervision, Project administration, Investigation, Funding acquisition. WB: Writing – review & editing, Visualization, Project administration.

## Footnotes

^1^ https://web.ExPASy.org/translate/

^2^ https://www.ebi.ac.uk/interpro/

^3^ https://web.expasy.org/protparam/

^4^ https://swissmodel.expasy.org/interactive

## Supplementary material

SUPPLEMENTARY FIGURE S1 The features of lncRNA sequencing, including **(A)** Exon number **(B)** Sequence length **(C)** Transcript number. **(D)** The length information of circRNA sequences from WTS data. **(E)** The length information of the circRNA ORF.

SUPPLEMENTARY FIGURE S2 Heatmap depicting the expression levels of genes from twelve WTS libraries based on the mean FPKM values, with blue and red colors representing downregulated and upregulated genes, respectively.

SUPPLEMENTARY FIGURE S3 The GO term analyses of DELs **(A–F)** for the host genes and target genes **(G–L)** among the six comparison groups. X-axis: gene numbers, Y-axis: different subcategories from three major functional categories of GO terms, including biological process (red), cellular component (blue), and molecular function (green). **(A,G)**: B vs. A, **(B,H)**: B vs., C, **(C,I)**: C vs. A, **(D,J)**: F vs. A, **(E,K)**: F vs. B, **(F,L)**: F vs. C.

SUPPLEMENTARY FIGURE S4 Top 20 KEGG pathways of DELs **(A–F)** for the host genes and target genes **(G–L)** among the six comparison groups. The colors and sizes of the circles represent the *q*-values and count numbers in different pathway enrichments, respectively. Rich Factor refers to the ratio of the number of differentially expressed genes located in this pathway term to the total number of genes located in this pathway term. X-axis: Rich Factor values, Y-axis: major enriched subcategories of KEGG pathway. **(A,G)**: B vs. A, **(B,H)**: B vs., C, **(C,I)**: C vs. A, **(D,J)**: F vs. A, **(E,K)**: F vs. B, **(F,L)**: F vs. C.

SUPPLEMENTARY FIGURE S5 The functional annotation of the host genes of DECs by GO enrichment among the six comparison groups **(A–F)**. Colors represent *p* values, and the circle size represent the count numbers in different pathway enrichments. X-axis: gene numbers, Y-axis: different subcategories from three major functional categories of GO terms, including biological process (red), cellular component (blue), and molecular function (green). Top 20 KEGG pathway enrichment analyses of the host genes of DECs among the six comparison groups **(G–L)**. The color and size of the circles represent the *q*-values and count numbers in different pathway enrichments, respectively. Rich Factor refers to the ratio of the number of differentially expressed genes located in this pathway term to the total number of genes located in this pathway term. X-axis: Rich Factor values, Y-axis: major enriched subcategories of KEGG pathway. **(A,G)**: B vs. A, **(B,H)**: B vs., C, **(C,I)**: C vs. A, **(D,J)**: F vs. A, **(E,K)**: F vs. B, **(F,L)**: F vs. C.

SUPPLEMENTARY FIGURE S6 Multiple sequence alignment of protein domain architecture from *R. pedestris* defensins and lysozymes. **(A)** defensins; **(B)** I-type lysozymes; **(C)** C-type lysozymes; **(D)** B-type lysozymes. The typical domains of all *R. pedestris* defensins and lysozymes are marked by red boxes. The consensus residues are marked by “*”, residues that are ≥ 75% identical among the aligned sequences are marked by “:”, and residues that are ≥ 50% identical among the aligned sequences are marked by “.”.

SUPPLEMENTARY FIGURE S7 The expression profiles of DE AMPs, DELs and DECs were estimated by qRT–PCR validation and WTS data among the six comparison groups. The gut samples were used for qRT–PCR validation from the remaining total RNA after library construction. Relative expression levels of qRT–PCR and the WTS were calculated using the log 2 of the relative quantification (RQ) values and log_2_ (Fold Change) values, respectively. Error bars indicate the mean ± standard deviation (*n* = 3).

SUPPLEMENTARY FIGURE S8 **(A)** The miRNA–AMP regulatory network. The red circles indicate AMP genes, and the green hexagons indicates miRNAs. **(B)** The putative secondary structures of novel-miRNA-154 and novel-miRNA-111. The purple nucleotides represent the sequence information of novel-miRNA-154-5p and novel-miRNA-111-5p.

SUPPLEMENTARY TABLE S1 The statistical information and mapping ratios of data quality from twelve lncRNA-Seq libraries.

SUPPLEMENTARY TABLE S2 The basic information and data quality of circRNA-Seq data from twelve libraries.

SUPPLEMENTARY TABLE S3 The prediction of internal ribosome entry site of circRNAs from WTS and annotation.

SUPPLEMENTARY TABLE S4 The sequence details were applied to construct phylogenetic trees of *defensin* and *lysozyme* family genes in this study. All these protein sequences are retrieved and download from NCBI Genbank.

SUPPLEMENTARY TABLE S5 Primers used for qRT-PCR validation in this study.

SUPPLEMENTARY TABLE S6 The Pearson correlation coefficients and *p* values of lncRNA-miRNA and miRNA-mRNA pairs for ceRNA construction.
