# Supplementary figures and images for: The ncRNA-mediated regulatory networks of defensins and lysozymes in Riptortus pedestris: involvement in response to gut bacterial disturbances
Source: Front Microbiol. 2024 May 17;15:1386345. doi: 10.3389/fmicb.2024.1386345 (PMC11140134; doi:10.3389/fmicb.2024.1386345)

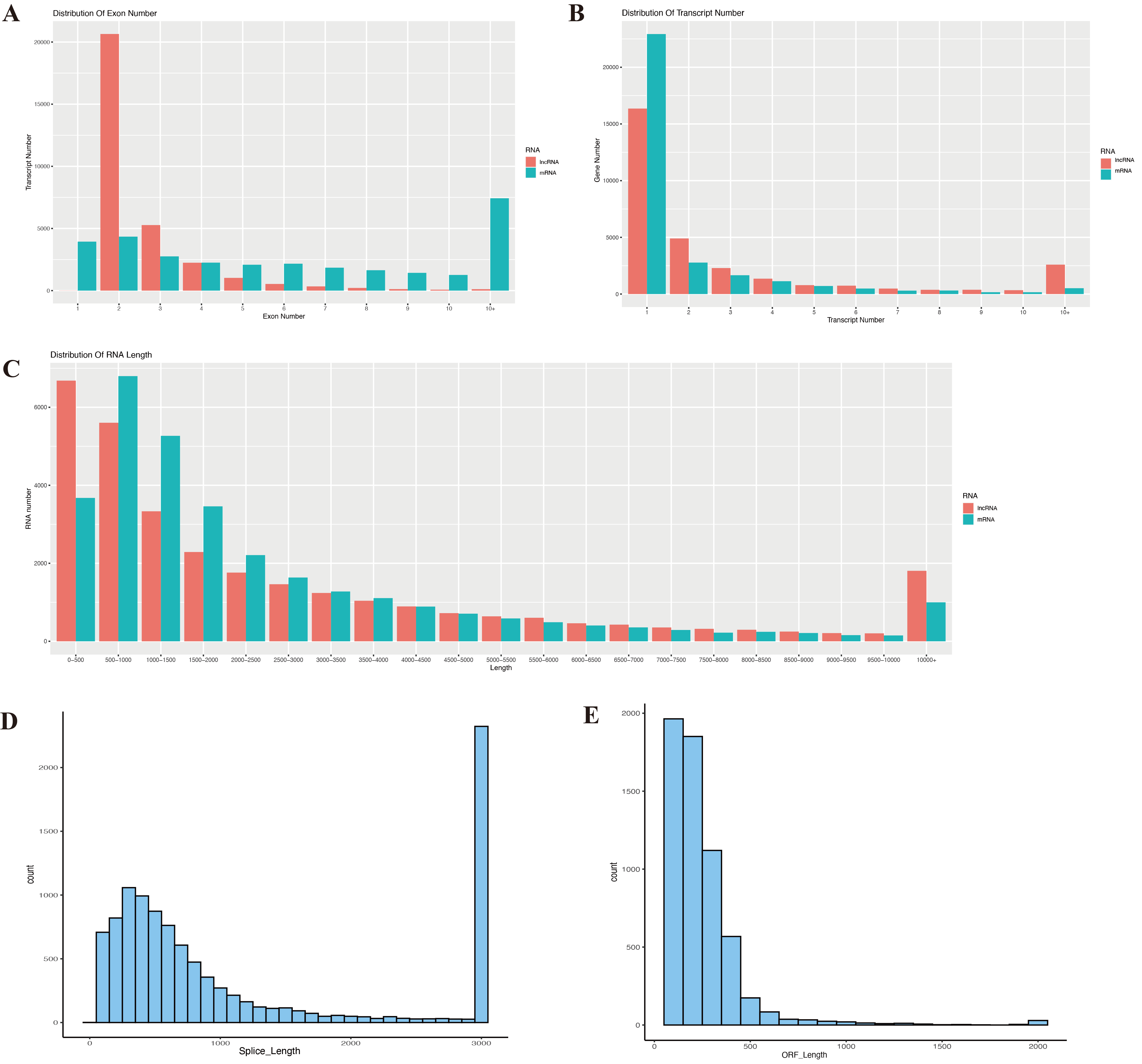

Supplement: SUPPLEMENTARY FIGURE S1 — The features of lncRNA sequencing, including (A) Exon number (B) Sequence length (C) Transcript number. (D) The length information of circRNA sequences from WTS data. (E) The length information of the circRNA ORF. [file Image_1.TIF]

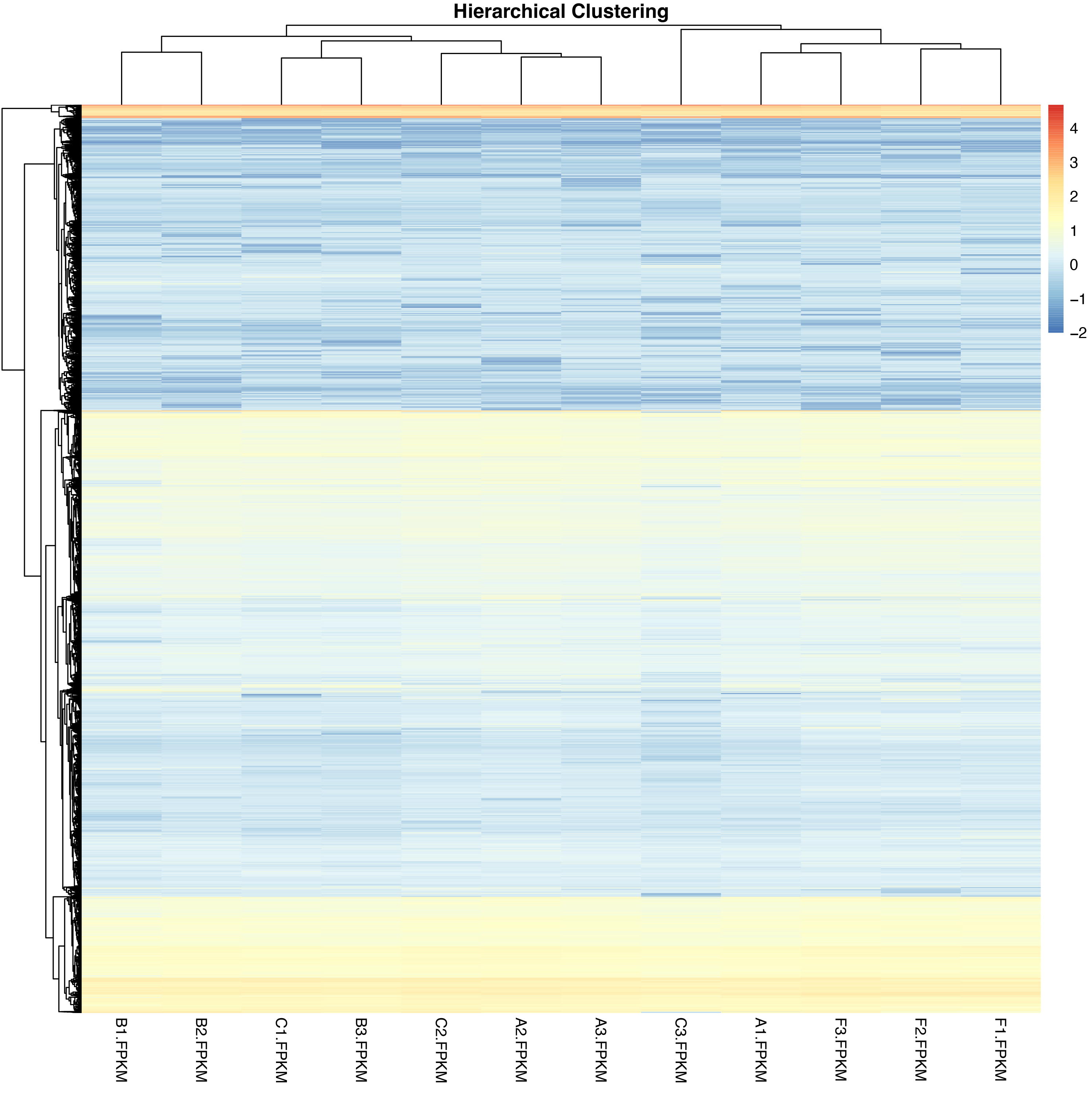

Supplement: SUPPLEMENTARY FIGURE S2 — Heatmap depicting the expression levels of genes from twelve WTS libraries based on the mean FPKM values, with blue and red colors representing downregulated and upregulated genes, respectively. [file Image_2.TIF]

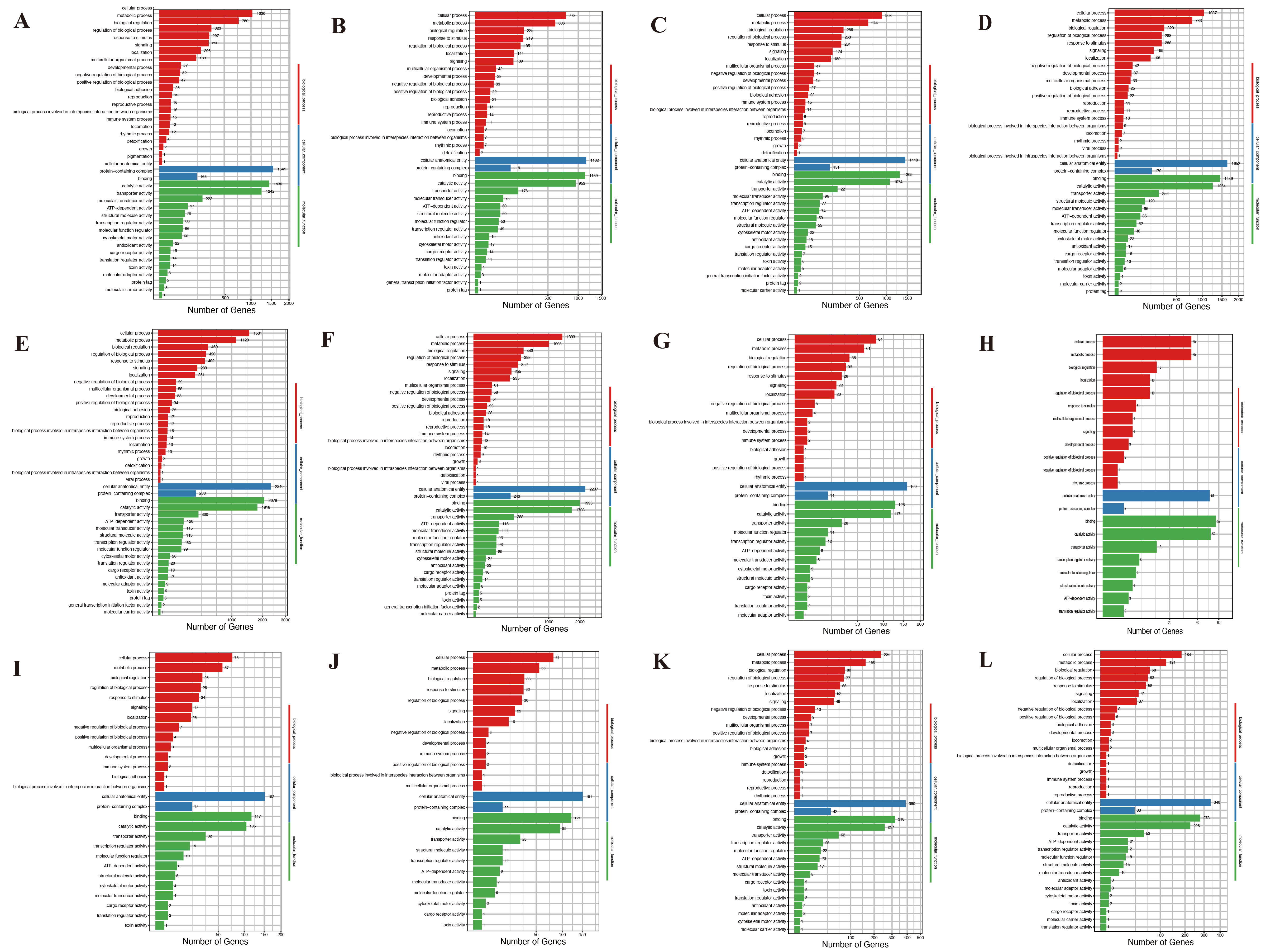

Supplement: SUPPLEMENTARY FIGURE S3 — The GO term analyses of DELs (A–F) for the host genes and target genes (G–L) among the six comparison groups. X-axis: gene numbers, Y-axis: different subcategories from three major functional categories of GO terms, including biological process (red), cellular component (blue), and molecular function (green). (A,G): B vs. A, (B,H): B vs., C, (C,I): C vs. A, (D,J): F vs. A, (E,K): F vs. B, (F,L): F vs. C. [file Image_3.TIF]

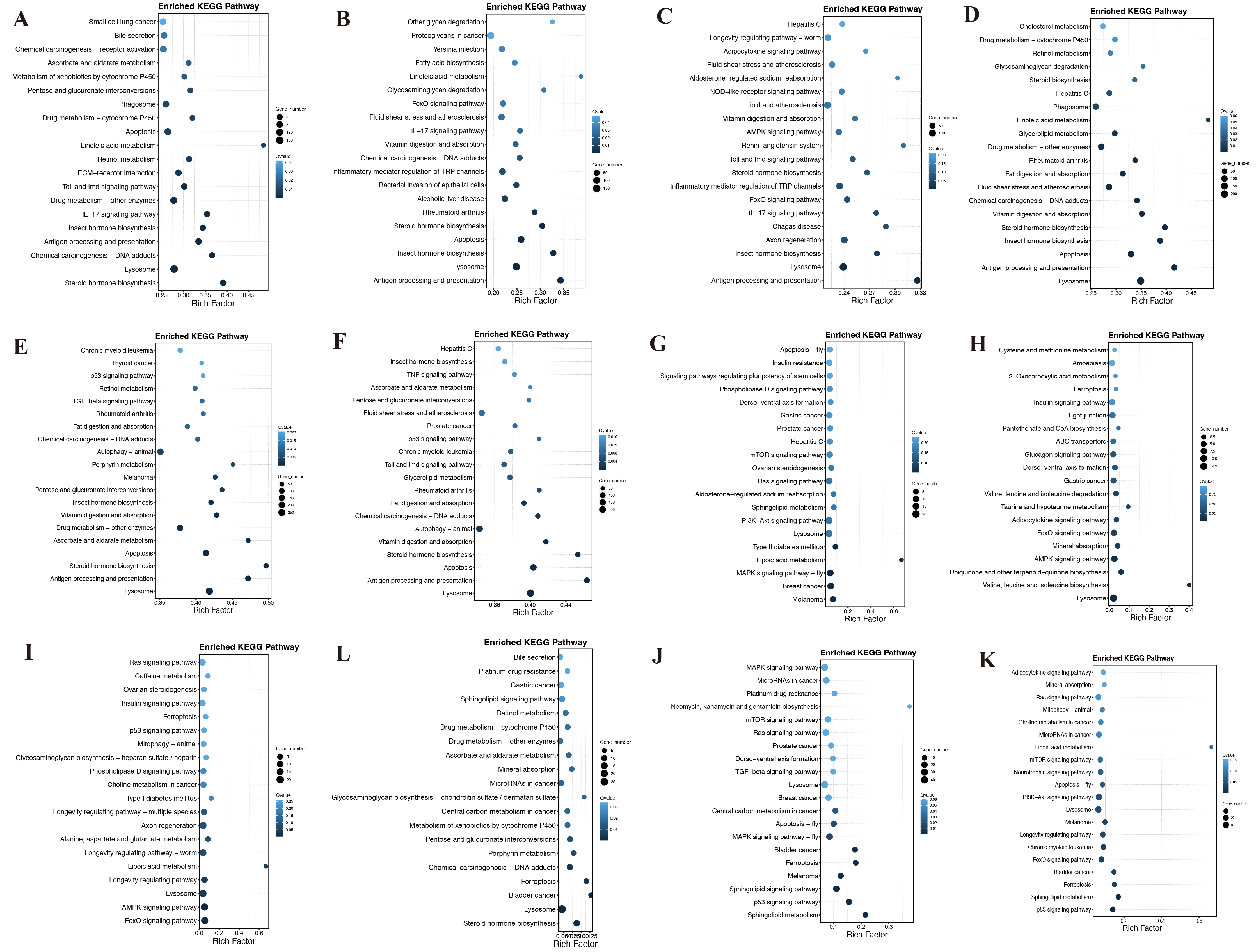

Supplement: SUPPLEMENTARY FIGURE S4 — Top 20 KEGG pathways of DELs (A–F) for the host genes and target genes (G–L) among the six comparison groups. The colors and sizes of the circles represent the q-values and count numbers in different pathway enrichments, respectively. Rich Factor refers to the ratio of the number of differentially expressed genes located in this pathway term to the total number of genes located in this pathway term. X-axis: Rich Factor values, Y-axis: major enriched subcategories of KEGG pathway. (A,G): B vs. A, (B,H): B vs., C, (C,I): C vs. A, (D,J): F vs. A, (E,K): F vs. B, (F,L): F vs. C. [file Image_4.TIF]

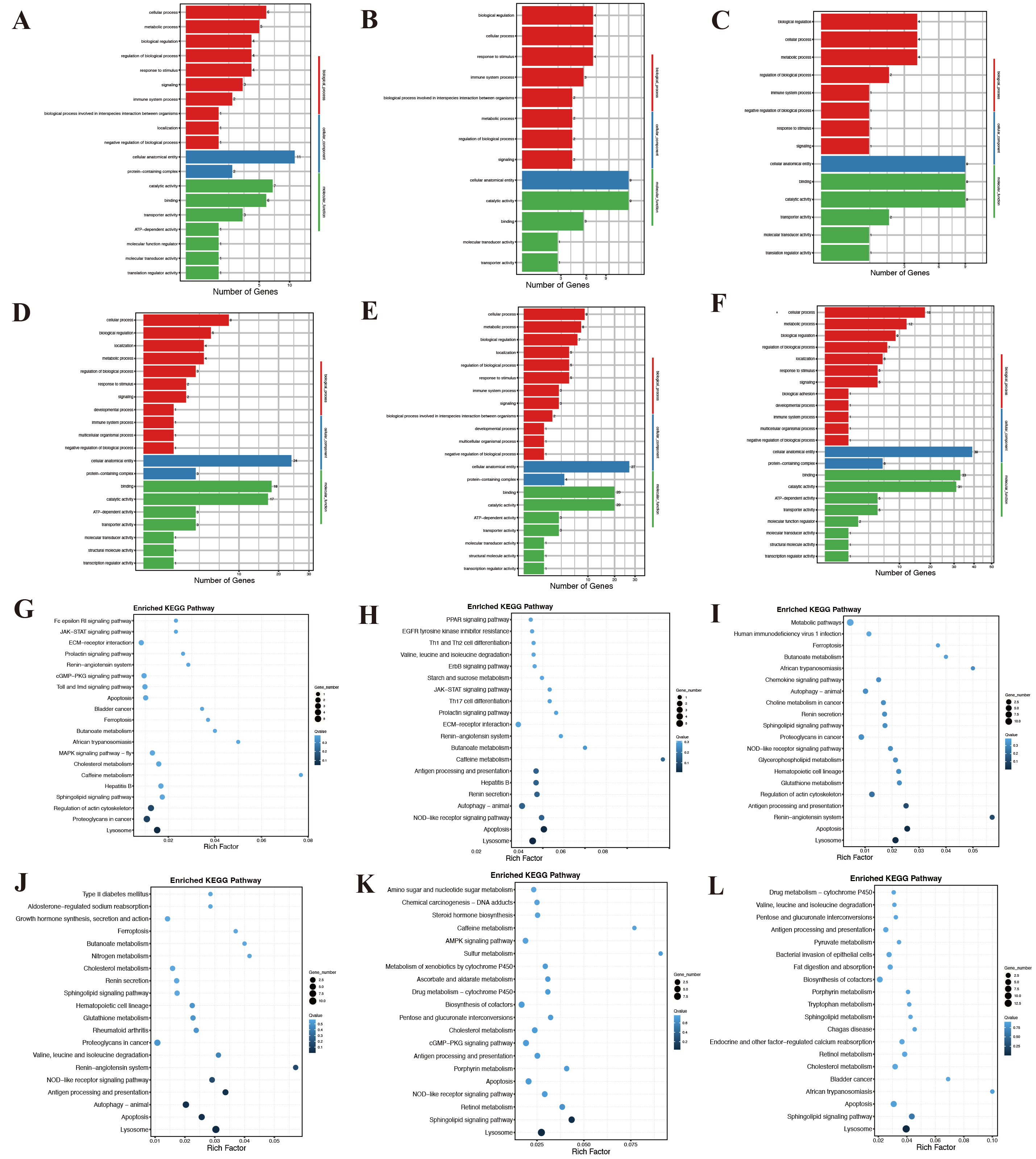

Supplement: SUPPLEMENTARY FIGURE S5 — The functional annotation of the host genes of DECs by GO enrichment among the six comparison groups (A–F). Colors represent p values, and the circle size represent the count numbers in different pathway enrichments. X-axis: gene numbers, Y-axis: different subcategories from three major functional categories of GO terms, including biological process (red), cellular component (blue), and molecular function (green). Top 20 KEGG pathway enrichment analyses of the host genes of DECs among the six comparison groups (G–L). The color and size of the circles represent the q-values and count numbers in different pathway enrichments, respectively. Rich Factor refers to the ratio of the number of differentially expressed genes located in this pathway term to the total number of genes located in this pathway term. X-axis: Rich Factor values, Y-axis: major enriched subcategories of KEGG pathway. (A,G): B vs. A, (B,H): B vs., C, (C,I): C vs. A, (D,J): F vs. A, (E,K): F vs. B, (F,L): F vs. C. [file Image_5.TIF]

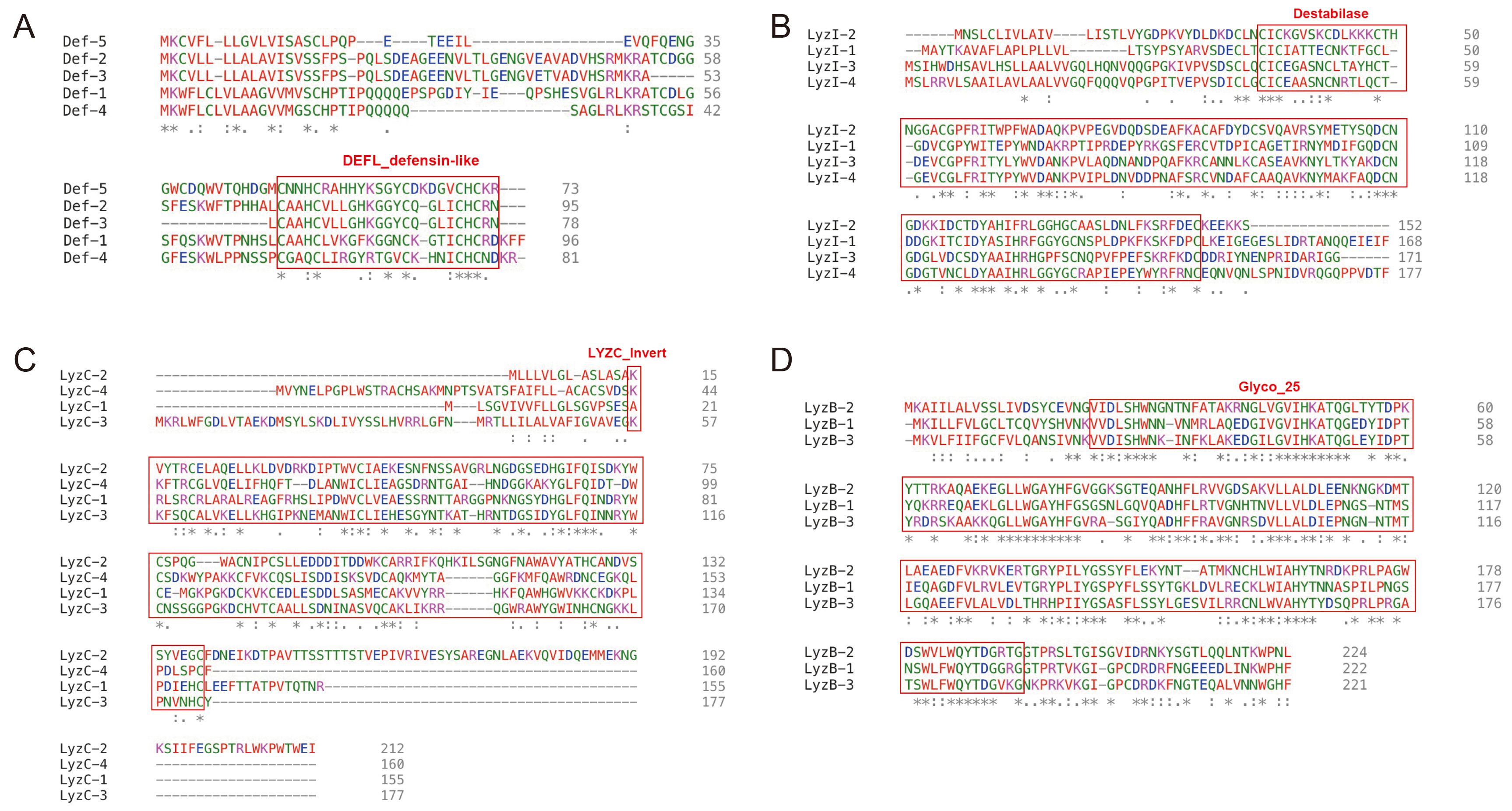

Supplement: SUPPLEMENTARY FIGURE S6 — Multiple sequence alignment of protein domain architecture from R. pedestris defensins and lysozymes. (A) defensins; (B) I-type lysozymes; (C) C-type lysozymes; (D) B-type lysozymes. The typical domains of all R. pedestris defensins and lysozymes are marked by red boxes. The consensus residues are marked by “*”, residues that are ≥ 75% identical among the aligned sequences are marked by “:”, and residues that are ≥ 50% identical among the aligned sequences are marked by “.”. [file Image_6.TIF]

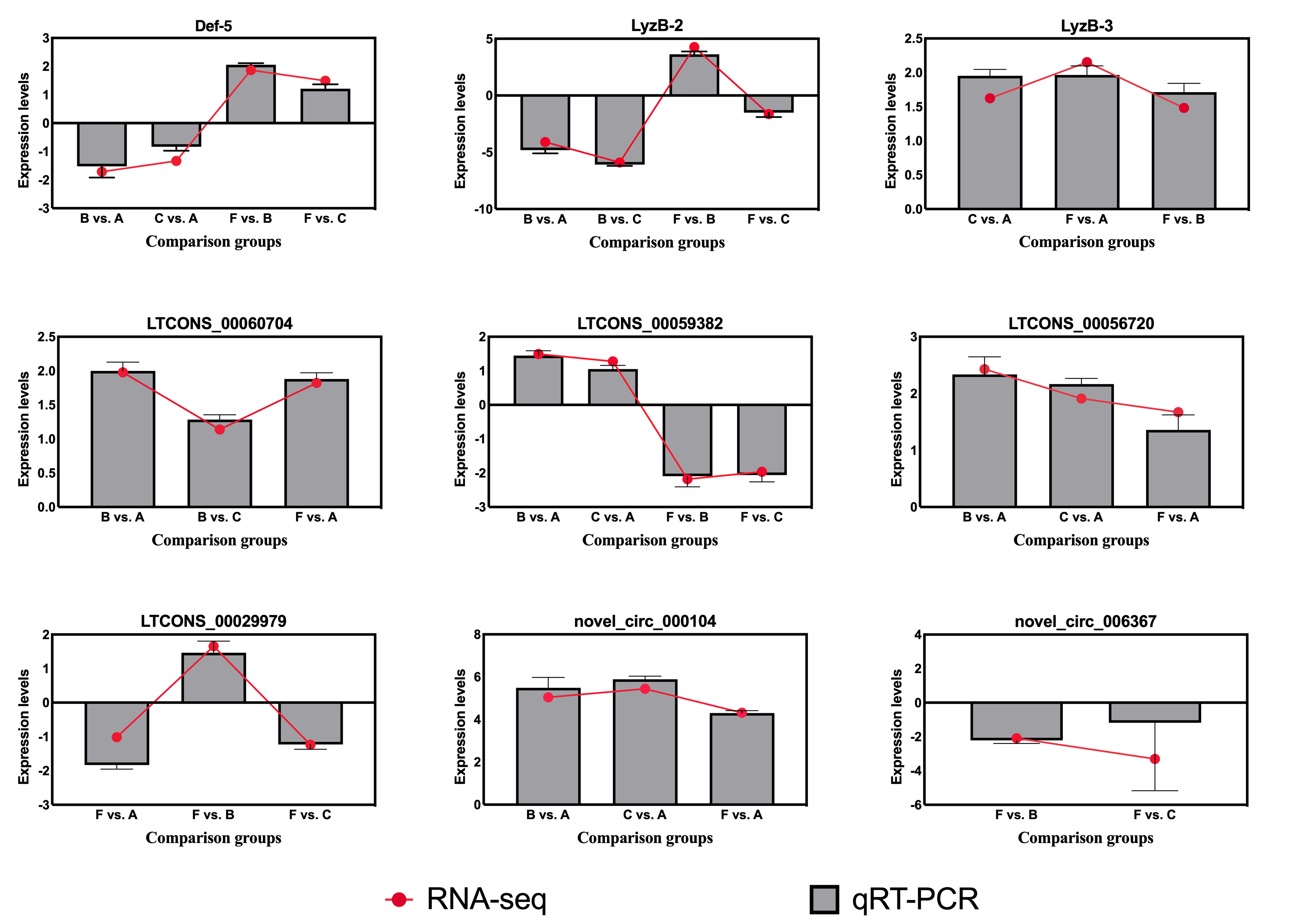

Supplement: SUPPLEMENTARY FIGURE S7 — The expression profiles of DE AMPs, DELs and DECs were estimated by qRT–PCR validation and WTS data among the six comparison groups. The gut samples were used for qRT–PCR validation from the remaining total RNA after library construction. Relative expression levels of qRT–PCR and the WTS were calculated using the log 2 of the relative quantification (RQ) values and log2 (Fold Change) values, respectively. Error bars indicate the mean ± standard deviation (n = 3). [file Image_7.TIFF]

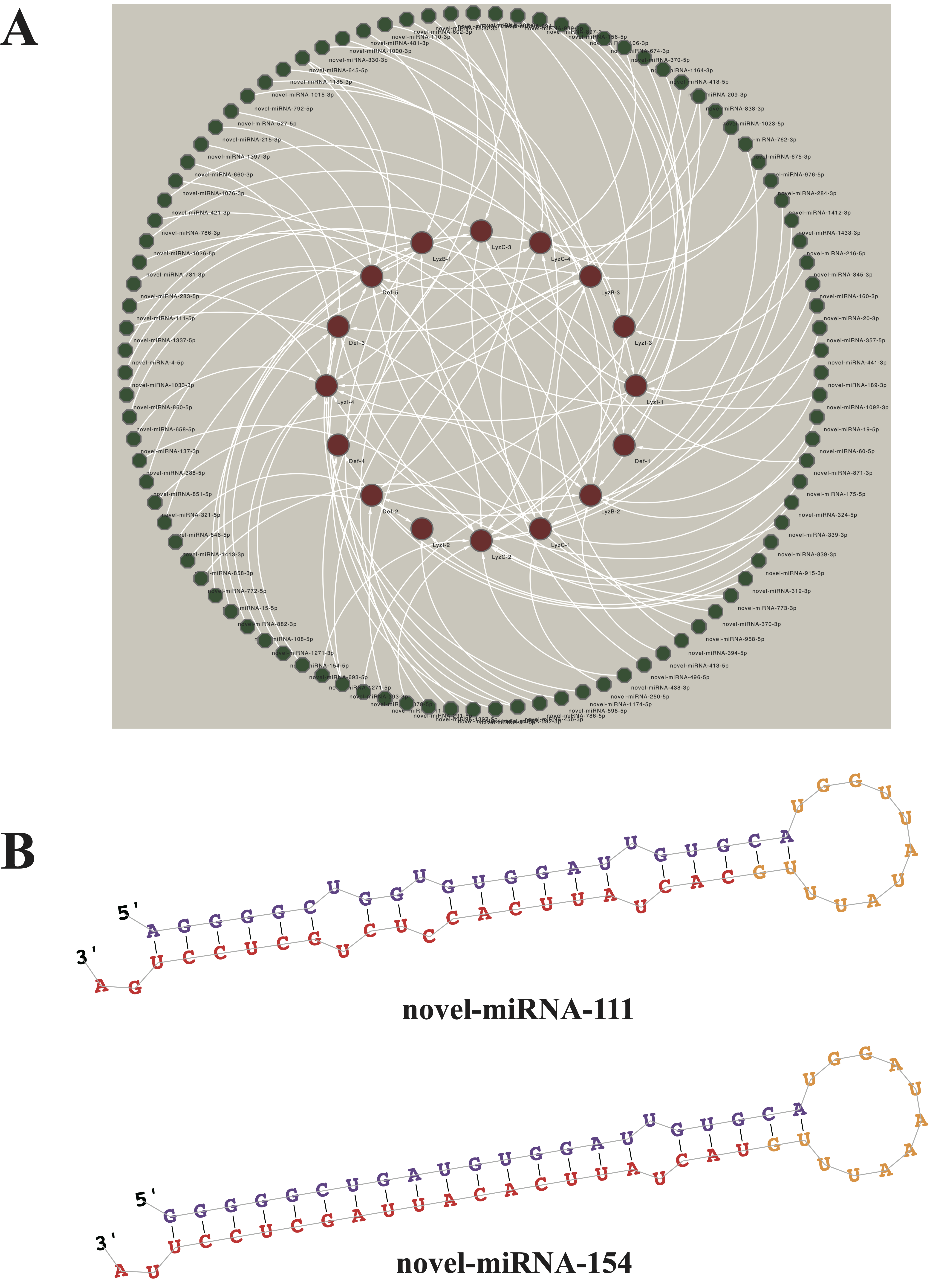

Supplement: SUPPLEMENTARY FIGURE S8 — (A) The miRNA–AMP regulatory network. The red circles indicate AMP genes, and the green hexagons indicates miRNAs. (B) The putative secondary structures of novel-miRNA-154 and novel-miRNA-111. The purple nucleotides represent the sequence information of novel-miRNA-154-5p and novel-miRNA-111-5p. [file Image_8.TIF]
